# Human umbilical cord mesenchymal stem cell delivery of mitochondria to melanocytes enhances skin repigmentation efficacy in autologous epidermal cell suspension transplantation through the TNFAIP2-TNT system

**DOI:** 10.7150/ijbs.128719

**Published:** 2026-01-30

**Authors:** Xiaodong Jin, Shishi Xiong, Bo Wang, Qi Wang, Jia Zhang, Zeqian Wang, Yuqi Zhou, Xiaoqi Chen, Tong Wu, Shanshan Gao, Chenxi Zhang, Qing Zhang, Yucheng Sun, Zhe Jian

**Affiliations:** Department of Dermatology, Xijing Hospital, Fourth Military Medical University, No. 127 of West Changle Road, Xi'an, Shannxi, 710032, China.

**Keywords:** Autologous epidermal cell suspension, Human umbilical cord mesenchymal stem cells, TNFAIP2-TNT system, Repigmentation, mitochondrial transfer

## Abstract

Autologous epidermal cell suspension (AECS) transplantation is essential for treating large-area depigmenting diseases and some chronic wounds that are refractory to conservative therapy. However, this technique encounters challenges, including limited repigmentation efficacy and high costs, which have hindered its clinical adoption. To enhance the repigmentation efficacy of AECS, our research explored the optimal conditions for preparing suspensions of epidermal cells (ECs). Next, we investigated the functions of melanocytes (MCs) and repigmentation efficacy through co-culturing and co-transplantation of human umbilical cord mesenchymal stem cells (hUCMSCs) with AECS. The addition of hUCMSCs to this system enhanced the proliferation, migration, and melanin synthesis capabilities of the ECs. These findings were validated *in vivo*, with the hUCMSCs co-transplantation group demonstrating superior skin repigmentation efficacy in mice. Then gene interference, overexpression, and transcriptome analyses were conducted to elucidate the mechanisms underlying the Tumor Necrosis Factor Alpha-Induced Protein 2 (TNFAIP2)-Tunneling Nanotubes (TNTs) system in mediating melanin synthesis. Our findings outline a novel pathway through which hUCMSCs, via the TNFAIP2-TNT system, mediate the delivery of mitochondria to melanocytes, enhancing the function of MCs. This presents new avenues for improving the repigmentation efficacy of AECS transplantation.

## Introduction

Autologous epidermal cell suspension (AECS) transplantation is essential for treating large-area depigmenting diseases, such as vitiligo and post-inflammatory hypopigmentation, as well as some chronic wounds that are refractory to conservative therapy 1-3. These facilities deliver a solution of live keratinocytes (KCs), melanocytes (MCs), fibroblasts, and Langerhans cells harvested from donor skin to the recipient site of transplantation, which can cover up to 80 times the area of the donor skin 4, 5. Nevertheless, these techniques are associated with challenges, including limited repigmentation efficacy, specialized skills, and high costs 5-7. Therefore, it is very necessary to develop a new method to enhance the repigmentation efficacy of AECS transplantation.

hUCMSCs, derived from umbilical cord matrix, are a type of pluripotent stem cell that possess functions such as regulating immunity, anti-apoptosis, and antioxidation, and are involved in tissue repair and regeneration 8. As mesenchymal stem cells (MSCs) can simultaneously activate several mechanisms, many researchers consider MSC transplantation to be the best and most effective approach in cell therapy 9. Previous studies have demonstrated that the co-culture of adipose-derived stem cells (ADSCs) with MCs enhances MCs proliferation and migration 10, and improves the efficacy of MCs transplantation in animal skin 11. Moreover, Lin, et al., observed that MSCs transferred mitochondria to endothelial cells under cellular stress, which enhanced endothelial cell engraftment 12. This phenomenon aligns with previous findings of transfer of mitochondria from MSCs to other cells, such as lung alveolar epithelial cells, neurons and macrophages, during tissue repair or metabolic remodel 13-15. However, the extent to which hUCMSCs enhance MCs and improve the repigmentation efficacy of AECS transplantation remains unclear.

In this study, we observed that hUCMSCs can form tunneling nanotubes (TNTs) with MCs to mediate mitochondrial transfer, which increases the intracellular ATP level in MCs. This, in turn, activates the PKA/MITF pathway, enhancing melanin synthesis. Additionally, it promotes the proliferation and migration of MCs, thereby improving the repigmentation efficacy of AECS transplantation. Our findings suggest a potential method to improve the treatment of depigmented skin diseases through the transplantation of AECS, offering a new direction for future research in exploring regenerative medicine and cell therapy.

## Materials and Methods

### The isolation and culturing of normal human epidermal cells (ECs)

Adult skin specimens obtained from circumcisions were used for the cultures, after obtaining approval from the Institutional Review Board of Xijing Hospital, Fourth Military Medical University (Approval number; XJLL-KY-20252156). The epidermis was separated from the dermis after treatment with 2.5 U/mL of Dispase II (17105041, Gibco, Grand Island, NY, USA) for 16 h. The epidermal sheets were treated with 0.125% trypsin for 15 minutes to produce a suspension of individual ECs. MCs were suspended in Medium 254 (M254500, Gibco, Grand Island, NY, USA) supplemented with 1× Human Melanocyte Growth Supplement-2 PMA-free (HMGS; S0165, Gibco, Grand Island, NY, USA) and 1× Penicillin-Streptomycin Liquid (PS; P1400, Solarbio, Beijing, China). KCs were suspended in EpiLife Medium (Calcium-Free; MEPICF500, Gibco, Grand Island, NY, USA) supplemented with 1× Human Keratinocyte Growth Supplement (HKGS; S0015, Gibco, Grand Island, NY, USA) and 1× PS. For all experiments, MCs at passages 2 through to 5 and KCs at passages 1 through to 2 were used.

### hUCMSCs preparation

Human umbilical cord mesenchymal stem cells, obtained from newborns' umbilical cords with the approval of the Institutional Review Board of Xijing Hospital, Fourth Military Medical University (Approval number; KY20242204). The cells were cultured in Mesenchymal Stem Cells Serum-free Medium (NC0103, Yocon, Beijing, China) supplemented with 1× MSC Serum Free Medium supplement 2 (NC0105.S, Yocon, Beijing, China) and 1× PS. For all experiments, MSCs at passages 5 through to 9 were treated with 5 μg/mL Mitomycin C (MMC; M5353, Sigma-Aldrich, USA) 37 ºC for 5 h before used.

### Co-culture of hUCMSCs with ECs

In direct co-culture, we counted 5×10^4^ MCs and 5×10^4^ hUCMSCs treated with MMC and inoculated in 6-well plates (703001, Nest, Beijing, China). In indirect co-culture, we inoculated 5×10^4^ MCs or KCs in the lower layer of 6-well plates and 5×10^4^ hUCMSCs in transwell chambers (TCS019006, Biofil, Guangzhou, China). The co-culture cells were cultured using Medium 254 supplemented with 1× HMGS or EpiLife Medium with 1× HKGS, and the medium was typically replaced every two days.

### Cell count and cell viability

The cells were counted by automatic cell counter, Countess® (Invitrogen). Cell viability was assessed by the addition of trypan blue dye 0.4% (15250061, Gibco, Grand Island, NY, USA) to 10 μL of the cell suspension, the number of stained (dead) and unstained (live) cells were counted.

### Flow cytometry

Cells were stained for flow cytometry and analysed with BD LSRFortessa™ (BD Biosciences, CA, USA) and FlowJo software (Tree Star). Antibody labelling was performed on ice for 20 min, followed by three washes with PBS (P1010, Solarbio, Beijing, China), and fixation with eBiosciencet^TM^ Foxp3/Transcription Factor Staining Buffer Set (00-5523-00, Invitrogen, Grand Island, NY, USA). Antibodies included APC anti-human CD117 (c-kit; 983302, Biolegend, USA), FITC anti-human CD90 (Thy1) Antibody (328108, Biolegend, USA), Zombie UV^TM^ Fixable Viability Kit (423107, Biolegend, USA). In the indicated experiments, cells that were directly co-cultured for one, two, and three weeks were dissociated using a trypsin/EDTA solution, 0.25% (T1300, Solarbio, Beijing, China), and then resuspended in PBS prior to flow cytometry analysis.

### Cell counting kit-8 (CCK-8) assay

The Cell Counting Kit-8 (GK10001, Glpbio, Shanghai, China) was utilized to assess the proliferation of co-cultured MCs and KCs. Following two weeks of indirect co-culture, the transwell chambers were removed, and the CCK8 reagent was mixed at a ratio of 1:10 with the corresponding medium and added to the bottom layer of the 6-well plate. The MCs were incubated at 37 ºC with 5% CO_2_ for 1.5 hours, while the KCs were incubated for 3 hours. Absorbance readings were taken at 450 nm using a microplate reader (Thermo Fisher Scientific, San Jose, CA).

### Transwell assay

The transwell assay was conducted using 24-well hanging cell culture inserts (PCEP24H48, Millipore, Bedford, MA). We inoculated 5×10^3^ MCs or a combination of 5×10^3^ MCs and 5×10^3^ hUCMSCs into the transwell chambers and subjected them to starvation treatment for 24 hours. Simultaneously, 5×10^4^ KCs were inoculated into the lower chamber with EpiLife Medium supplemented with 1× HKGS. Following a 72-hour incubation period, we removed the culture medium and rinsed the transwell chambers with PBS. The polyethylene terephthalate (PET) membrane was then treated with Masson-Fontana Melanin Staining Solution (DJ0021, LEAGENE, Beijing, China), causing the migrated melanocytes to stain black. Finally, we utilized an inverted microscope to capture the results.

### Scratch assay

The MCs or KCs in the logarithmic growth phase were collected and inoculated 5×10^5^ on the lower chamber. Upon reaching 70-80% cell confluence, the cells were scratched and treated with MMC. The experimental group was placed in the upper chamber with hUCMSCs for co-cultured. After 72 hours, we used an inverted microscope to observe the migration conditions.

### Immunofluorescence staining

After co-culturing for two weeks, the cells were seeded at a density of 2×10^4^ in a confocal dish. They were then incubated at 37 ºC with 5% CO_2_ for 24 hours before being fixed with 4% paraformaldehyde (PFA; G1101-500ML, Servicebio, Wuhan, China). Following fixation, the cells were permeabilized using 0.1% Triton X-100 in PBS and blocked for 30 minutes with 5% bovine serum albumin (BSA; 9048-46-8, Biofroxx). Incubation with primary antibodies, such as Anti-Melan-A antibody (ab210546, Abcam, UK, 1:1000), Anti-TRP1 (ab235447, Abcam, UK, 1:1000), DCT Monoclonal antibody (68114-1-Ig, Proteintech, USA, 1:1000), Alexa Fluor® 647 Anti-E Cadherin Antibody (ab194982, Abcam, UK, 1:600), Anti-N Cadherin antibody (ab19348, Abcam, UK, 1:600), Alexa Fluor® 647 Anti-Cytokeratin 10 antibody (ab194231, Abcam, UK, 1:500), and Anti- Cytoskeleton 5 Marker (ab321868, Abcam, UK, 1:1000), was performed at 4 ºC for 16 hours. This was followed by three washes with PBST and a 1-hour incubation with goat anti-rabbit or anti-mouse secondary antibody at room temperature. The cells were then washed three times with PBST and covered with Fluoroshield^TM^ with DAPI, liquid (F6057, Sigma-Aldrich, USA) for 5 minutes. Finally, the confocal laser scanning microscope (LSM 880, Zeiss, was used to observe the dishes.

### Melanin content

The cells that were directly co-cultured with PBS were washed and then dissociated using 0.25% trypsin/EDTA. Then counted 1×10^5^ cells treated with solution contained 1 M NaOH and 10% DMSO and subjected to a water bath at 80 ºC for 30 minutes. Absorbance readings were taken at 450 nm to detect the absorbance of the supernatant. Relative melanin synthesis growth rate (%) was defined as the ratio of melanin content (OD) measured in 1×10^5^ cells before and after co-cultured for one week.

### Tyrosinase activity

To assess tyrosinase activity, the cells that were directly co-cultured were washed with PBS and dissociated using 0.25% trypsin/EDTA, after which they were resuspended in PBS. We then counted 1×10^5^ cells treated with the Tyrosinase Activity Test Kit (BC4050, Solarbio, Beijing, China), and aspirated 100 μL of the supernatant to detect the absorbance at 505 nm. Relative melanin synthesis growth rate (%) was defined as the ratio of tyrosinase activity (U/1×10^6^ cells) measured in 1×10^5^ cells before and after co-cultured for one week.

### Animal studies

8-week-old female BALB/c nude mice (GemPharmatech, Nanjing, China) were used in this study. Following a one-week acclimatization period, the animals were anesthetized via an intraperitoneal injection of Tribromoethanol (M2960, Nanjing Aibei Biotechnology Co., Ltd, China) and inhalation of isoflurane. Subsequently, the mice were marked on an area of approximately 1×1 cm² on the dorsal lateral back using a pen. Dermabrasion was performed using a small felt wheel attached to a hand-piece motor (Saeshin Strong 90, Korea), operated at 5,000 rpm. A rubber ring was applied to immobilize the sanded area and prevent the surrounding epidermis from migrating into the wound. To enhance grafting efficiency, endothelial cells (ECs) alone and in combination with hUCMSCs were mixed with Matrix-Gel™ Basement Membrane Matrix (C0372-5ml, Beyotime, Shanghai, China) before application to the dermabraded area. After 5 minutes, the area was covered with saline-soaked gauze, secured with sticking plaster, and reinforced with self-adhesive tape.

### Microscopic analysis

The mice were euthanized, and the skin from the transplant site was excised and submerged in 10% buffered formalin. Subsequently, the skin was embedded in paraffin blocks. The paraffin-embedded skin samples were sliced into sections 4 μm thick and stained with hematoxylin and eosin (H&E). The Brightfield Sweep Analysis was conducted using a Slideview (OLYMPUS VS200, Japan).

### Masson-Fontana assay

After dewaxing the paraffin sections, washed them once with PBS, then stained with Fontana Silver Ammonia solution at room temperature in the dark for 16 hours. Subsequently, rinsed with double-distilled water, and dropwise stained with hypo solution at room temperature for 10 minutes. Then, dropwise stained with neutral red solution at room temperature for 5 minutes. After washing, mounted the sections and analyzed with Slideview.

### Colorimetric Analysis

Skin color was measured using a 9NBO X82 spectrophotometric colorimeter (9nbo, Suzhou Jiulian Technology Co., Ltd., China) before transplantation (baseline L*) and at the 21^st^ day post-transplantation. The L* value was recorded at three different points within the transplant area, and the average value was calculated for each time point. Then calculated ΔL* according to baseline L* and post-transplantation L* value.

### Immunohistochemistry (IHC)

In the third week following cell transplantation, the transplant sites were harvested, fixed in formalin, and embedded in paraffin blocks. Immunohistochemistry (IHC) was performed to identify the colonized mast cells (MCs). The slides were dewaxed, antigens were retrieved, and then treated with 3% hydrogen peroxide in PBS for 10 minutes at room temperature to block endogenous peroxidase activity. After blocking nonspecific binding with 5% normal goat serum (NS02L, Sigma-Aldrich, USA), the slides were incubated with primary antibodies, Anti-Melan A antibody (ab210546, Abcam, UK, diluted 1:1000), for 18 hours at 4˚C. The sections were washed with PBS three times and then incubated for 30 minutes with Goat Anti-Rabbit IgG H&L (HRP; ab6721, Abcam, UK, diluted 1:1000) secondary antibodies. DAB color development solution was applied for 5 minutes at room temperature, and then color development was terminated with distilled water. This was followed by hematoxylin restaining of cell nuclei for 5 minutes, gradient dehydration, xylene transparency, and brightfield sweep analysis with Slideview.

### TUNEL assay

Apoptosis was measured using the CoraLite® Plus 488 TUNEL Assay Apoptosis Detection Kit (PF00006, Proteintech, USA). All samples were washed twice with double distilled water, followed by the addition of Proteinase K solution, diluted in 100 µl PBS, which was applied dropwise to each sample for 10 minutes. After washing with PBS, 50 µl of TUNEL reaction solution was added to each sample, and the samples were incubated in the dark at 37 °C for 1 hour. Finally, the slices were washed three times with PBS containing 0.1% Triton X-100 and 5 mg/ml BSA to reduce background noise, and the FITC channel of the confocal laser scanning microscope was used for observation.

### RNA sequencing analysis

RNA sequencing was conducted by Gene Denovo Biotechnology Co., Ltd. (Guangzhou, China). Total RNA was extracted using TRIzol reagent (Invitrogen, Grand Island, NY, USA), following the manufacturer's protocol. RNA quality was determined on a NanoDrop 2000 spectrophotometer (Thermo Fisher Scientific, USA). Gene expression levels were quantified utilizing fragments per kilobase of transcript per million fragments mapped (FPKM). Bioinformatic analysis was performed using Omicsmart, a dynamic real-time interactive online platform for data analysis (http://www.omicsmart.com).

### MCs isolation from co-culture system

After co-culturing directly with hUCMSCs, MCs were resuspended in autoMACS Running Buffer-MACS Separation Buffer (130-091-221, Miltenyi Biotec, Germany). Subsequently, hUCMSCs were isolated using CD105 MicroBeads for humans (130-051-201, Miltenyi Biotec, Germany), and the cells obtained through negative selection were identified as MCs.

### Scanning electron microscope (SEM) observation

1×10^4^ MCs and hUCMSCs were inoculated onto cell culture slides, respectively, and the medium was discarded after co-culturing at 37 °C for 8 hours. The slides were washed three times with PBS, placed in Electron Microscope Fixative (G1102-100ML, Solarbio, Beijing, China) at 4 °C for 24 hours, and the cellular connectivity was observed using scanning electron microscopy.

### *In vitro* mitochondrial transfer by co-culture

We incubated MitoTracker™ Red FM (M22425, Thermo Fisher Scientific, Waltham, MA, USA) with hUCMSCs, as well as MitoTracker™ Green FM (M7514, Thermo Fisher Scientific, Waltham, MA, USA) with MCs, for 30 minutes at 37 °C. Subsequently, the MTR-hUCMSCs were co-cultured with the MTG-MCs at 37 °C for 8 hours to observe mitochondrial transmission between the two cell types.

### ROS assay

MCs were treated with hUCMSCs, Nocodazole (18.75 nM, HY-13520, MCE, New Jersey, USA), or Cytochalasin D (18.75 nM, HY-N6682, MCE, New Jersey, USA) for 72 hours. Total cellular ROS was measured using the Reactive Oxygen Species Assay Kit (S0033S, Beyotime, Beijing, China), following the manufacturer's instructions.

### ATP measurement

MCs were treated with hUCMSCs, Nocodazole, or Cytochalasin D for 72 hours. Total cellular ATP was measured using the Enhanced ATP Assay Kit (S0027, Beyotime, Beijing, China) according to the manufacturer's instructions, with lumnescence detection.

### Apoptosis assay

MCs were treated with hUCMSCs, Nocodazole, or Cytochalasin D for 72 hours. Apoptosis was assessed using FITC-conjugated Annexin V and propidium iodide (PI) staining (Annexin V-FITC/PI Apoptosis Kit; E-CK-A211, Elabscience, Wuhan, China) via flow cytometry, following the manufacturer's instructions.

### JC-1 assay

MCs treated with hUCMSCs, Nocodazole, or Cytochalasin D were evaluated using the Enhanced Mitochondrial Membrane Potential Assay Kit with JC-1 (C2003S, Beyotime, Beijing, China), following the manufacturer's instructions. The fluorescent intensities for the aggregate (red FL, 590 nm) and monomeric (green FL, 530 nm) forms of JC-1 were measured at Ex/Em = 490/530 nm and 525/590 nm using a microplate reader and a confocal laser scanning microscope. The percentage of membrane potential was represented as the FL590/FL530 ratio.

### Plasmids and siRNA

*TNFAIP2* overexpression (*TNFAIP2*-OE) plasmids were created by cloning the corresponding cDNAs into the pcDNA3.1 vector. The siRNA oligonucleotides used were: siNC: sense: 5'-UUCUCCGAACGUGUCACGUTT-3'; antisense: 5'-ACGUGACACGUUCGGAGAATT-3'.

si*TNFAIP2*: sense: 5'-UGUCGGAGGCCUCCUCUGATT-3'; antisense: 5'-UCAGAGGAGGCCUCCGACATT-3'.

*TNFAIP2*-OE plasmids, as well as gene-specific siRNA (50 nM) were mixed with Lipofectamine 3000 (Thermo Fisher Scientific, Waltham, MA, USA), and then transfected MCs for 24 h.

### Transcription-quantitative polymerase chain reaction (RT-qPCR)

The expression levels of the relevant genes were quantitatively assessed by analyzing their mRNA transcripts. The total RNA from human MCs was extracted using the FastPure Cell/Tissue Total RNA Isolation Kit V2 (RC112, Vazyme, Nanjing, China), following the manufacturer's protocol. cDNA was reverse transcribed from 1 μg of RNA using the Prime Script^TM^ RT reagent Kit (RR037Q, TaKaRa, Japan), and RT-qPCR was conducted with the TB Green Premix Ex Taq (RR420Q, TaKaRa, Japan). The 2^-△△CT^ method was employed to determine the relative mRNA expression levels.

### Western blot analysis

The cells were washed with ice-cold PBS and lysed in RIPA Lysis Buffer (P0013C, Beyotime, Beijing, China) containing Phenylmethanesulfonyl fluoride (PMSF; ST505, Beyotime, Beijing, China) at 4 ºC. After centrifugation at 12,000 rpm for 15 minutes, the protein concentration was quantified using a Pierce BCA Protein Assay Kit (E-BC-K318-M, Elabscience, Wuhan, China), and equal amounts of protein were separated on XPAGE 4-12% Bis-Tris Protein Gels (X15412LGel, ACE Biotechnology, Nanjing, China). Membranes were blocked for 1 hour at room temperature with 5% skim milk in 0.1% Tween/TBS. They were then incubated overnight at 4 ºC with the following antibodies: anti-TNFAIP2 (ab155256, Abcam, UK, 1:1000), anti-TUBA1B (ab108629, Abcam, UK, 1:100000), anti-F-actin (ab130935, Abcam, UK, 1:500), anti-TOMM20 (ab186735, Abcam, UK, 1:5000), anti-cAMP (ab76238, Abcam, UK, 1:50000), anti-CREB (ab32515, Abcam, UK, 1:1000), anti-p-CREB (ab32096, Abcam, UK, 1:5000), anti-Tyrosinase (ab170905, Abcam, UK, 1:2000), Anti-MITF (ab303530, Abcam, UK, 1:1000), Anti-TRP1 (ab235447, Abcam, UK, 1:1000), Anti-DCT (ab221144, Abcam, UK, 1:1000), Anti-Melan-A (ab210546, Abcam, UK, 1:1000), and anti-GAPDH (ab8245, Abcam, UK, 1:10000). Following incubation, the membranes were washed three times in 0.1% Tween/TBS and subsequently incubated for 1 hour at room temperature with the appropriate secondary antibody: Goat anti-Mouse IgG/HRP or Goat anti-rabbit IgG/HRP antibody (1:5000).

### Statistical analysis

All experiments, including representative micrographs, are independently repeated a minimum of three times, and all data are presented as the mean ± standard deviation. Statistical significance was determined using a two-tailed Student's *t*-test, One-way ANOVA with Bonferroni's post-test analysis, or two-way ANOVA analysis: *P≤0.05, **P≤0.01, ***P≤0.001, ****P≤0.0001 indicated significance, while ns (*P*>0.05) denoted no significant difference. Statistical analyses and graphical representations were performed using GraphPad Prism version 10 software (GraphPad, San Diego, CA, USA).

## Results

### The optimal conditions for preparing epidermal cell (EC) suspensions

**Figure 1:**
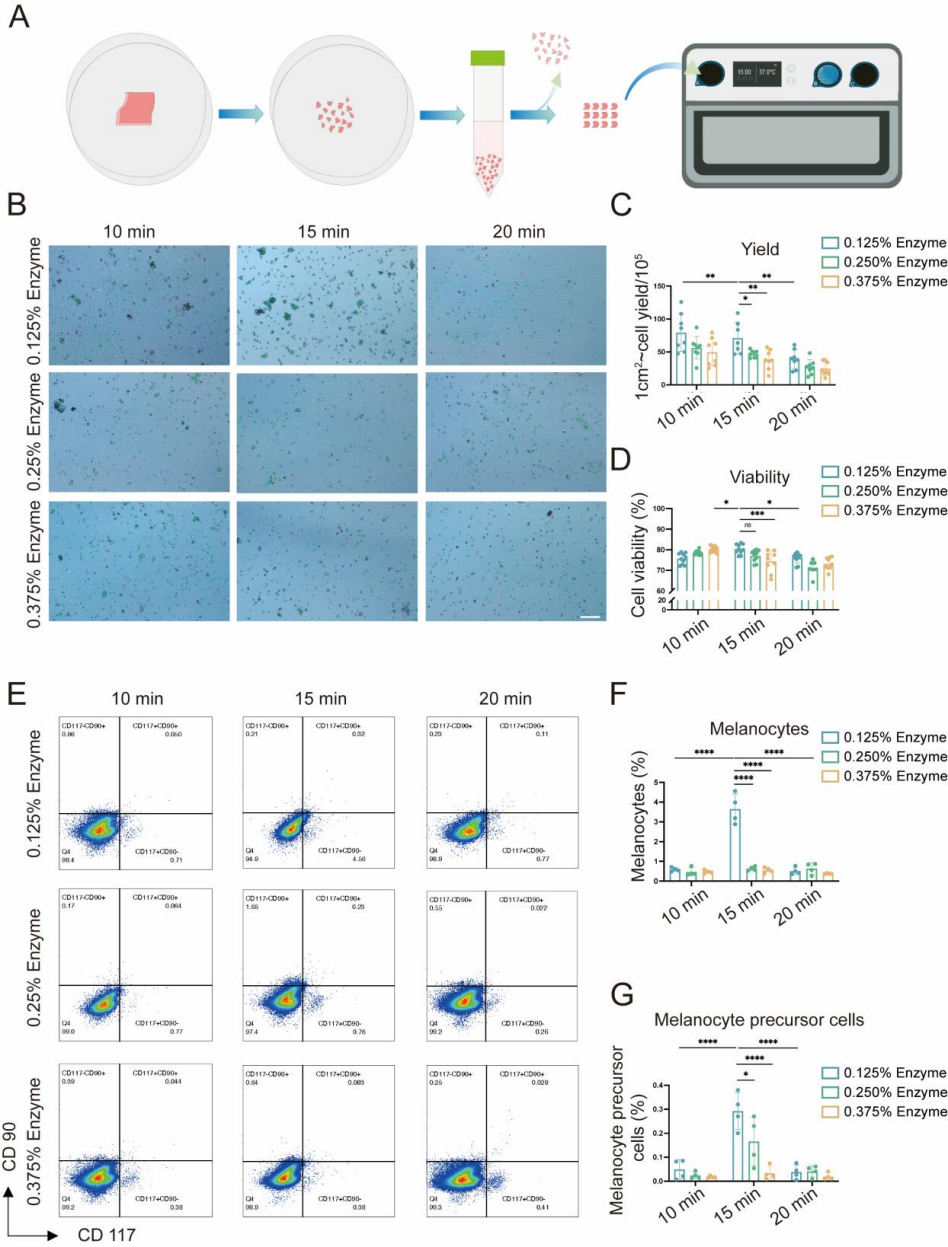
** Explore the optimal preparation conditions of epidermal cell (EC) suspensions. (A)** Flow chart for the application of disposable autologous epidermal separator for the preparation of ECs suspensions. **(B)** ECs of different preparation conditions were stained with Trypan blue and then observed with the cell counter, with live cells labelled in green and dead cells labelled in red. Scale bar: 200 μm. **(C-D)** Statistical analysis on cell viability and cell yield respectively. **(E)** hCD117 and hCD90 identified by flow cytometry; hCD117+hCD90- identifies MCs and hCD117+hCD90+ identifies melanocyte precursor cells. **(F-G)** Statistical analysis on melanocytes and melanocyte precursor cells respectively. Data represent mean ± S.D, and experiments were repeated a minimum of three times. *P≤0.05, **P≤0.01, ***P≤0.001, ****P≤0.0001, by two-way ANOVA analysis.

Cell viability and yield are key factors influencing the efficacy of AECS grafting 7, 16, 17. To enhance its therapeutic effect, we initially explored the optimal preparation conditions for AECS, including trypsin concentrations (0.125%, 0.250%, 0.375%) and digestion times (10, 15, 20 minutes). This was achieved using a disposable epidermal separator equipped with constant temperature heating and vibration functions (Zhende Medical Co., Ltd., Shaoxing, China) (Fig. 1A). Then we detected the cell yield and survival rate of the prepared AECS. The results indicated that the epidermal cell suspension, prepared through digestion with 0.125% trypsin at 37 °C for 15 min, exhibited a higher cell yield and survival rate (Fig. 1B-D). Furthermore, it was discovered that both mature MCs and melanocyte precursor cells constituted the highest proportion in the AECS prepared under the condition of 0.125% trypsin digestion for 15 min (Fig. 1E-G) 18. Based on the aforementioned studies, digestion with 0.125% trypsin for 15 min can maximally dissociate MCs from the basal part of the epidermis while maintaining high cellular activity.

### The presence of human umbilical cord-derived mesenchymal stem cells (hUCMSCs) enhances the function of epidermal cells (ECs)

**Figure 2:**
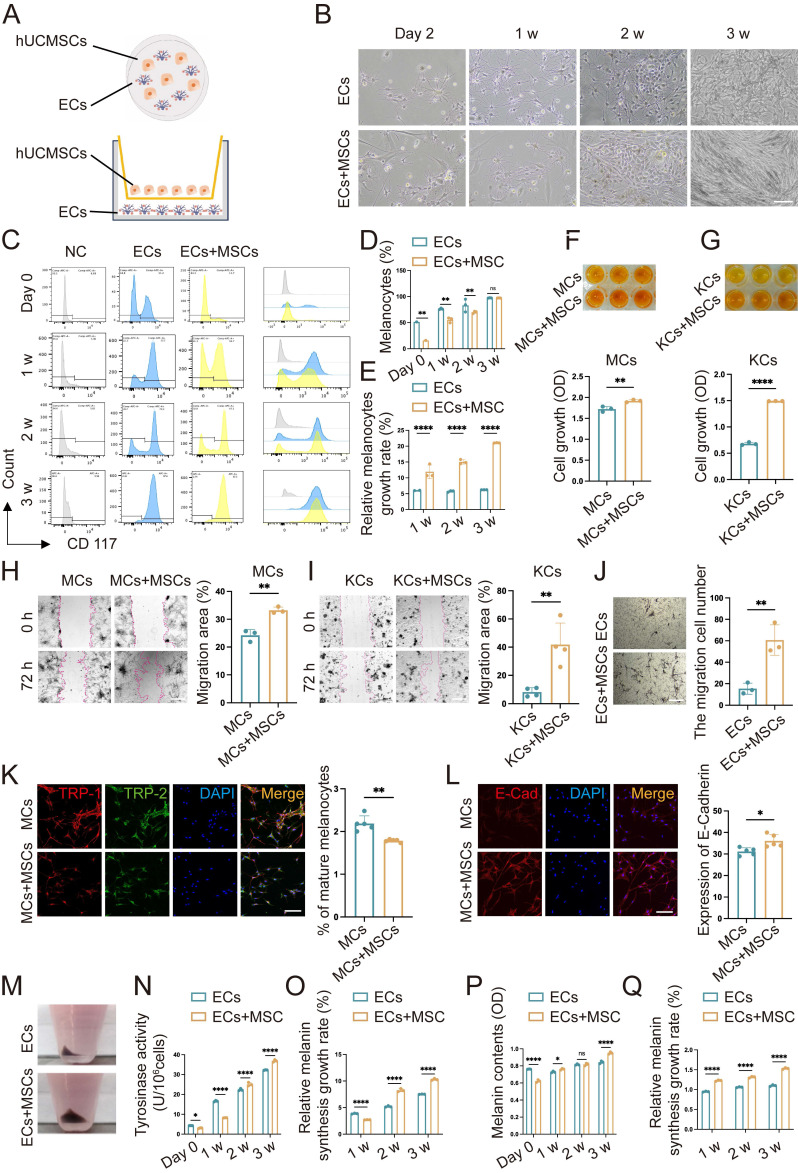
** Co-culture with hUCMSCs enhances the function of ECs. (A)** Diagram of direct co-culture (dir. co) and indirect co-culture (ind. co) process. **(B)** Observation of ECs alone and ECs+MSCs (dir. co) by light microscope. Scale bars: 100 μm. **(C)** Flow cytometry of MCs in ECs comparison of ECs alone and ECs+MSCs (dir. co). **(D)** Statistical analysis of MCs ratio in ECs and ECs+MSCs at week 1, week 2 and week 3. **(E)** Counted and calculated the total number of the absolute number of MCs, then compared it with the baseline number to determine the proliferation rate of MCs. **(F-G)** Proliferative ability of MCs and KCs (ind. co) at week 2 by CCK-8. **(H-I)** Migration ability of MC and KCs compared with hUCMSCs co-cultured (ind. co) at 72 h. Scale bars: 200 μm. **(J)** Transwell assay on ECs and ECs+MSCs (dir. co) with masson-fontana to identify MCs. Scale bars: 100 μm. **(K-L)** Immunofluorescent staining of MCs alone and MCs + hUCMSCs at week 2 suggesting the differentiation situation and adhesion ability of MCs. Scale bars: 30 μm. **(M)** Digested and collected the cells after culturing ECs alone and ECs+MSCs for three weeks. **(N)** Tyrosinase activity of ECs alone and ECs + hUCMSCs after co-cultured for 1 w, 2 w and 3 w.** (O)** The ratio of tyrosinase activity measured in 1×10^5^ cells before and after co-cultured for one week. **(P)** Melanin content of 1×10^5^ ECs alone and ECs + hUCMSCs after co-cultured for 1 w, 2 w and 3 w. **(Q)** The ratio of melanin content measured in 1×10^5^ cells before and after co-cultured for one week. Data show mean ± S.D, and each experiment was conducted with a minimum of three replicates. *P≤0.05, **P≤0.01, ****P≤0.0001, by two-tailed Student's *t* test.

Previous studies have shown that the proliferation and migration of melanocytes are significantly stimulated by co-culture with adipose-derived stem cells (ADSC), compared with melanocyte monocultures. However, the effect of this stimulation is limited 10. hUCMSCs exhibit greater proliferation and differentiation capabilities than ADSCs, and possess superior immune regulatory properties, indicating a stronger therapeutic potential 8. Therefore, we investigated the effects of co-culturing with hUCMSCs on ECs using both direct and indirect co-culture methods (Fig. 2A). We established three groups: one with ECs alone, another with hUCMSCs alone, and co-culture groups with varying ratios of ECs to hUCMSCs. We observed that MCs in the co-culture group, inoculated at a ratio of 1:1, exhibited a relatively fast proliferation rate (Fig. S1). Consequently, the 1:1 ratio was selected for the subsequent experiments within the co-culture group.

Next, we inoculated MMC-treated hUCMSCs and ECs into a co-culture system and observed them under a light microscope weekly for a period of three weeks. It was observed that after two to three weeks, the number of ECs in the co-culture group had increased significantly (Fig. 2B). The numerical change in MCs was examined using flow cytometry with CD117 antibody. It was observed that after three weeks of co-culture, the proportion of MCs in both groups nearly reached 100%. We measured the cell counts of each group and calculated the MCs proliferation rate using the absolute value of MCs (Fig. S2). The results indicated that the rate of MC proliferation in the co-culture group was higher than that in the MCs alone group at all time points (Fig. 2C-E). To avoid the interaction between MCs and KCs, we conducted Cell Counting Kit-8 (CCK8) experiments on them co-cultured with hUCMSCs respectively following indirect co-culture for a duration of two weeks. The results indicated that the enhancement of MCs proliferation ability by hUCMSCs in our study involves both direct contacting induced material transfer and changes in biological signals, as well as indirect effects of certain cytokines released by the former (Fig. 2F-G). As the main component cells of ECs, we observed the effects of hUCMSCs on the migration ability of MCs and KCs respectively. The former plays a major role in the repigmentation of the transplant sites, while the latter is crucial for wound closure and assists in the engraftment of MCs indirectly 19.

It was observed that MCs and KCs co-cultured with hUCMSCs exhibited a stronger migratory ability. (Fig. 2H-I). Considering the interaction between KCs and MCs, we conducted a transwell assay on co-cultured ECs and hUCMSCs. Masson-Fontana staining was conducted to identify the migrated MCs. It was observed that ECs co-cultured with hUCMSCs exhibited a stronger migratory ability (Fig. 2J). TRP-1 is expressed exclusively in mature MCs, whereas DCT is expressed in all MCs. Therefore, the detection of TRP-1/DCT indicates the proportion of mature MCs 9. The results indicated that in the presence of hUCMSCs, MCs could maintain a preferable stemness (Fig. 2K). Additionally, we observed that ECs co-cultured with hUCMSCs exhibited higher E-Cadherin expression, indicating a stronger adhesion function (Fig. 2L). The melanin synthesis capacity of MCs is closely associated with the repigmentation condition following AECS grafting 20-22. We found that ECs co-cultured with hUCMSCs exhibited a higher melanin synthesis capacity and melanin levels, suggesting that hUCMSCs could enhance the melanin synthesis capacity of MCs (Fig. 2M-Q).

In conclusion, the presence of hUCMSCs enhances the proliferation, migration, and adhesion functions of ECs, with MCs exhibiting a stronger melanin synthesis ability. These results suggest that co-culturing with hUCMSCs can improve the function of MCs.

### Co-transplantation with hUCMSCs enhances skin repigmentation efficacy in mice

**Figure 3:**
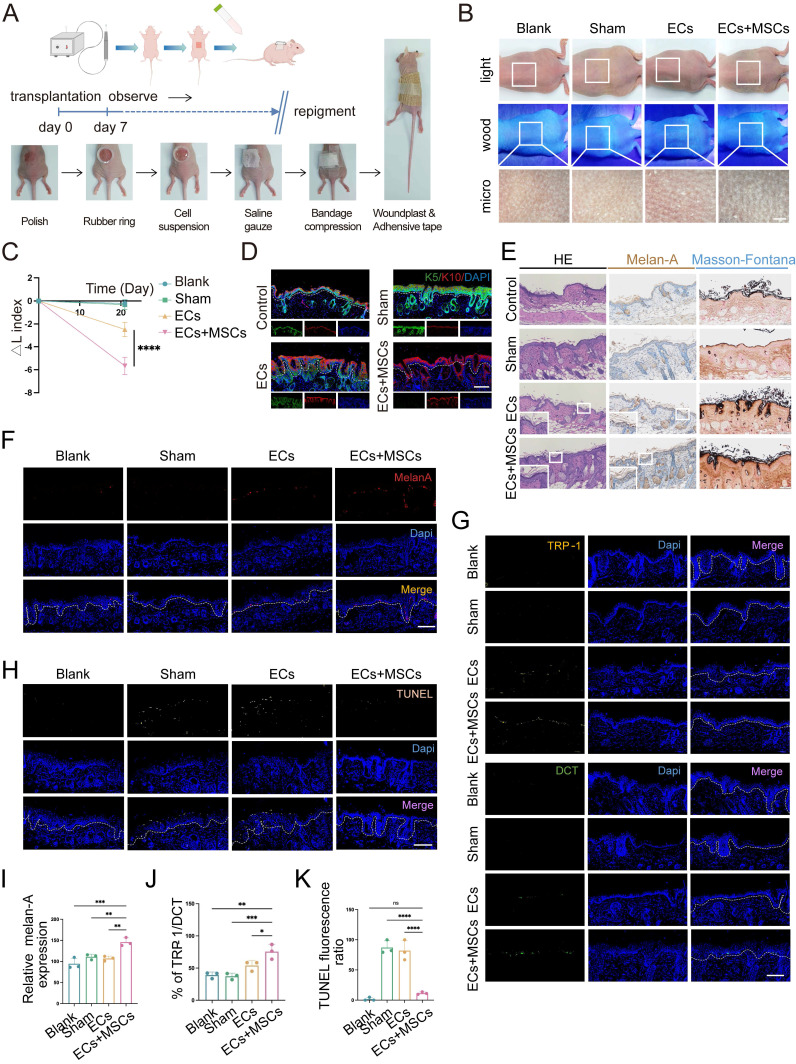
** Co-transplantation of hUCMSCs enhances the function of ECs. (A)** Diagram of transplanting the prepared AECS onto the polished back of nude mice process. **(B)** After transplantation for 21 days, the transplantation sites were observed under white light, wood light, and skin microscope. Scale bars: 500 μm. **(C)** Changes in skin pigmentation were assessed spectrophotometrically and ΔL values were quantified before and after animal transplantation. **(D)** After transplantation, the source of ECs in the corresponding sites were represented by human reactive K10 (hu-K10) and human/mouse reactive K5 (hu/ms-K5). Scale bars: 100 μm. **(E)** Light microscopy of skin specimens stained with H&E, Melan-A immunohistochemistry, and Fontana-Masson to compare the residing location of MCs and melanin content. Scale bars: 100 μm. **(F-H)** Immunofluorescence staining: Skin specimens stained with Melan-A, TRP-1, DCT and TUNEL apoptosis assay to detect the number, mature proportion, and apoptosis of MCs. Scale bars: 100 μm. **(I-K)** Semi-quantitative analysis of the fluorescence intensity. Data show mean ± S.D, and each experiment was conducted with a minimum of three replicates. *P≤0.05, **P≤0.01, ***P≤0.001, ****P≤0.0001, ns indicates no significant difference (n=3; one-way ANOVA with Bonferroni's post-test analysis).

To validate the aforementioned results *in vivo*, we utilized a BALB/c nude mouse model characterized by immune deficiency to receive cell suspension transplantation. Drawing upon the *in vitro* findings that the optimal growth conditions and melanin synthesis levels of ECs, we transplanted a mixture of AECS and hUCMSCs at the same 1:1 ratio in the co-transplantation group (Fig. 3A). Next, we transplanted cell suspensions and ensured the cell suspension remained within the local tissue. Then started to observe the transplanted sites until repigmentation (Fig. S3).

After 21 days, the skin pigmentation in transplantation sites were observed under white light, wood light, and skin microscope. It showed that the mice in the AECS and hUCMSCs co-transplantation group had a larger dark area on the back, and the corresponding areas were observed under skin microscope with darker color (Fig. 3B). To quantify the pigmentation intensity of transplanted area, we assessed the changes in skin pigmentation with ΔL values. The results confirmed that the ΔL values were consistent with visual observations (Fig. 3C). We then stained the skin samples with a human-reactive K10 antibody and a human/mouse-reactive K5 antibody to determine the origin of the ECs at the transplant sites. The results indicated that the ECs in both transplant groups were predominantly of human origin, whereas the ECs in the blank and sham groups were of mouse origin, suggesting successful transplantation (Fig. 3D). We then conducted hematoxylin and eosin (H&E), Melan-A immunohistochemistry, and Fontana-Masson staining on mouse skin samples to compare and observe the distribution of MCs and melanin synthesis in the transplanted skin. The findings indicated that the co-transplantation of hUCMSCs led to a greater colonization of MCs in the basal layer of the mouse skin (Fig. 3E). To objectively compare the MC count in each group of skin samples, we utilized melan-A fluorescence staining and conducted a range of fluorescence intensity area statistics to represent the relative MC colonization. The findings indicated that the hUCMSCs co-transplantation group exhibited a higher MC colonization (Fig. 3F, I). To further examine the function of colonized MCs, we conducted TRP-1/DCT staining on skin samples. Then we calculated the ratio of the two fluorescence intensities of the same animal to represent the proportion of mature MCs with melanin synthesis function. The findings revealed that the proportion of mature MCs was higher in the hUCMSCs co-transplantation group (Fig. 3G, J). Finally, TUNEL staining was conducted on the skin samples, and the cellular status at the transplantation sites was examined. The findings indicated that apoptotic cells were more prevalent in the skin of mice from both the sham group and the ECs transplantation group, whereas the number of apoptotic cells in the skin of mice from the hUCMSCs co-transplantation group was notably decreased (Fig. 3H, K).

In summary, the presence of hUCMSCs enhances the engraftment of transplanted ECs in the recipient's skin and inhibits their apoptosis, thereby improving the functionality of the ECs.

### Co-culture of MCs with hUCMSCs upregulated genes associated with cytoskeleton and induced tunneling nanotubes (TNTs) formation

**Figure 4:**
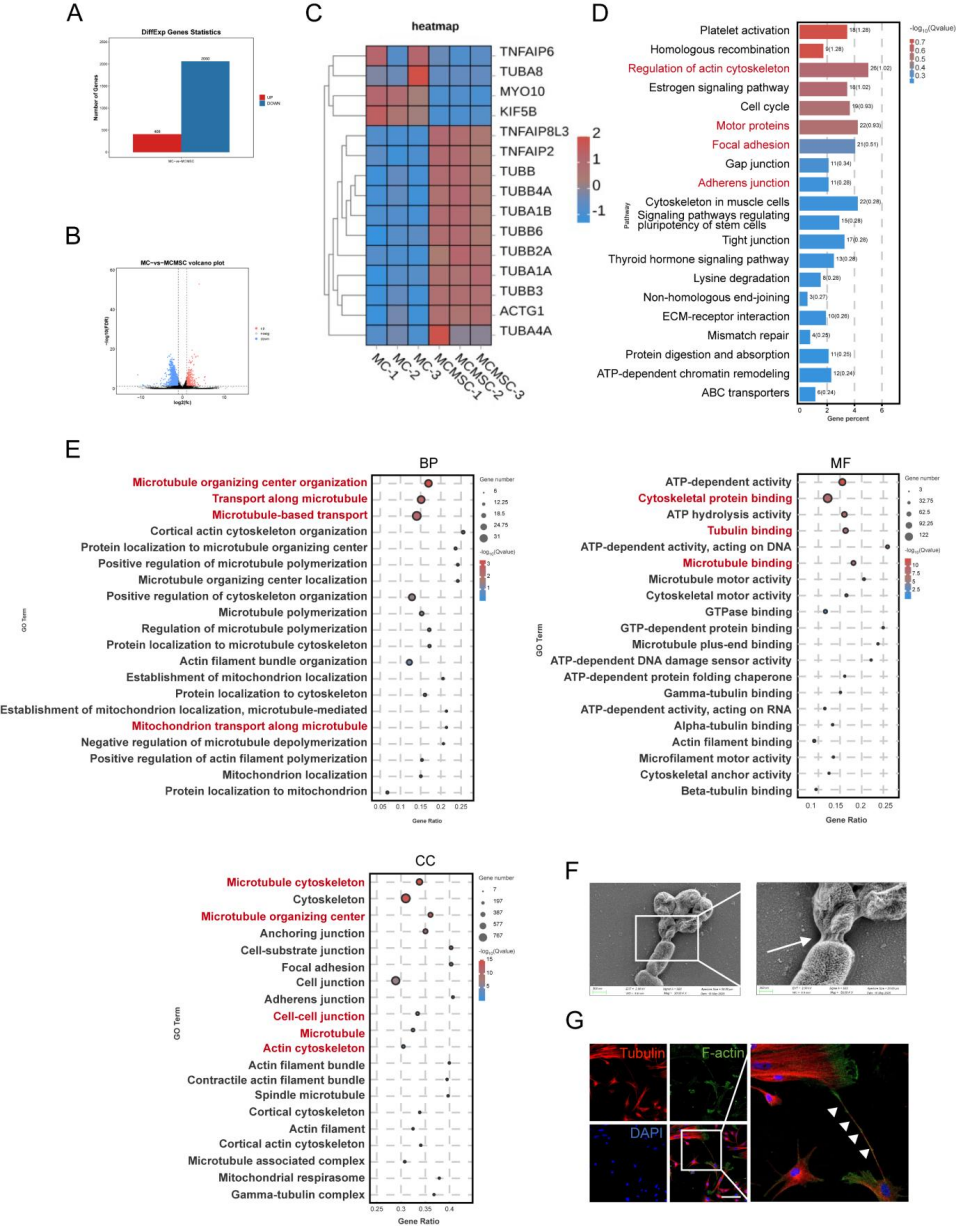
** hUCMSCs co-culture enhances cytoskeleton and cell-cell connection pathway. (A-B)** Histogram and Volcano plot depicted differential expressing genes of MC samples: MCs co-cultured with hUCMSCs (co-MCs) versus MCs alone (MCs).** (C)** Differential gene analysis heatmap: co-MCs versus MCs.** (D)** Bulk RNA-seq of MC samples: KEGG analysis (positive enrichment) for co-MCs versus MCs. **(E)** GO analysis (positive enrichment) for co-MCs versus MCs. **(F)** Captured the connections between cells with SEM. Scale bars: 500nm, 200nm. **(G)** Immunofluorescent staining cell-cell connection between hUCMSCs and MCs was showed using TNTs compositions, Tubulin and F-actin. Scale bars: 30 μm.

To gain insights into the mechanism by which hUCMSCs facilitate the transplantation of AECS, we conducted RNA sequencing (RNA-seq) analysis. Globally, there were 408 upregulated genes and 2060 downregulated genes across the two groups (Fig 4A-B). Our study suggested that the expression of TNTs-related genes was upregulated after co-cultured with hUCMSCs by differential gene analysis (Fig. 4C).

To gain further insight into the transcriptional differences, we conducted Kyoto Encyclopedia of Genes and Genomes (KEGG) enrichment analysis. Notably, hUCMSCs co-cultured with MCs exhibited significant enrichment in genes associated with the regulation of the cytoskeleton and cell junctions, compared to MCs cultured alone (Fig. 4D). Consistent with Gene Ontology (GO) enrichment analysis, hUCMSCs co-cultured with MCs exhibited enrichment in genes associated with components of the cytoskeleton, transport related to microtubules, and various cell junctions compared with MCs alone (Fig. 4E). This may be associated with the regulation of TNTs formation between MCs and hUCMSCs, which mediates the transfer of certain substances such as mitochondria, endosomes, and lysosomes, and have been identified in various tissues and cell types 23-27. Therefore, we utilized scanning electron microscopy (SEM) to examine the cellular and we observed cell connections resembling TNTs extending from the cytoskeleton, with diameters ranging from 50 to 700 nm (Fig. S4A) 28. As the morphology and size were different, we recognized the TNTs between hUCMSCs and MCs (Fig. 4F). As the components of TNTs are filamentous actin (F-actin) and microtubules, we utilized F-actin and tubulin staining to identify the structure of TNTs connecting two cells 29, 30. A slender tubular structure was observed between the two types of cells, with both F-actin and tubulin markers present simultaneously (Fig. 4G).

### TNTs mediate mitochondrial transfer from hUCMSCs to MCs and enhance MC function

**Figure 5:**
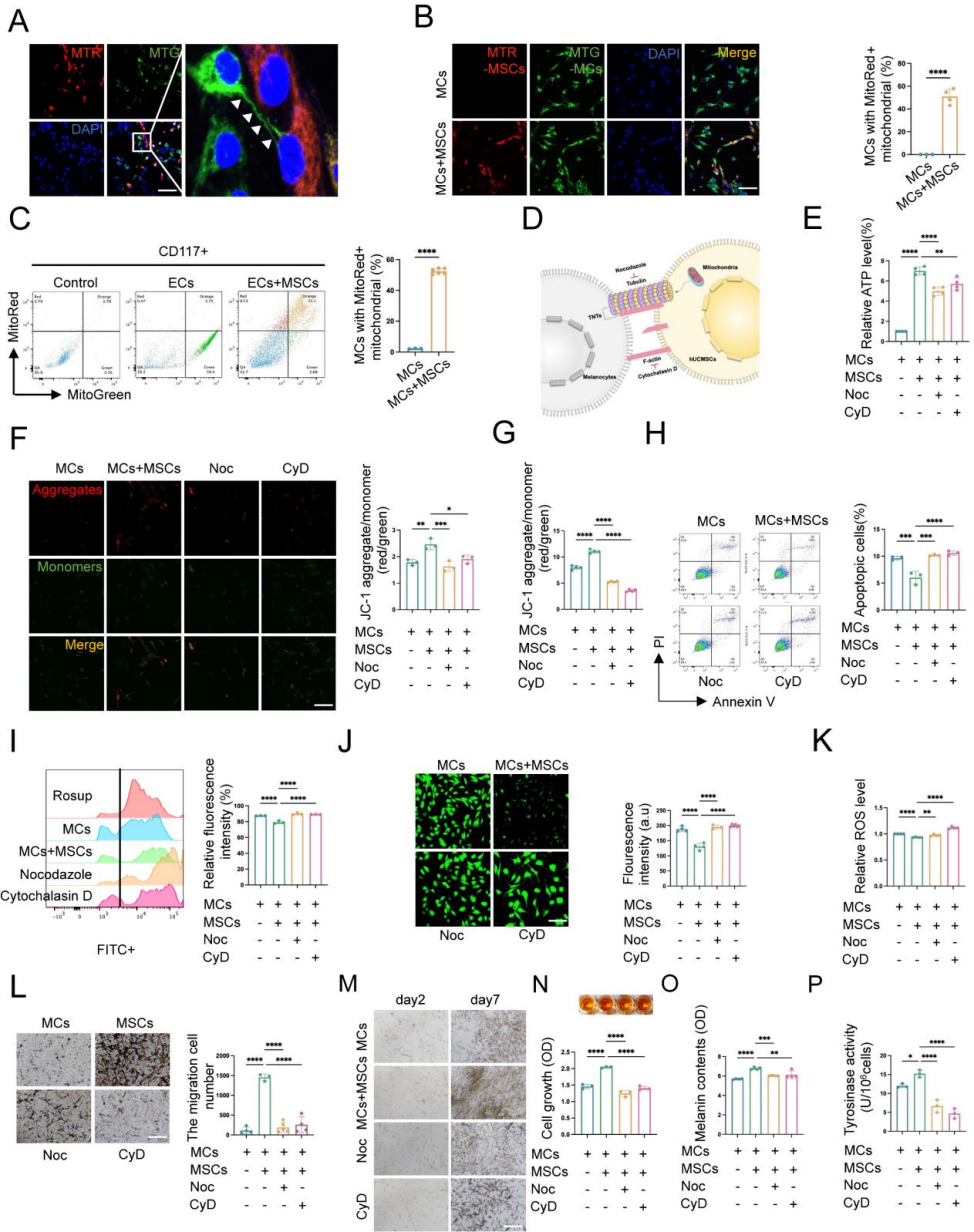
** hUCMSCs enhance MCs function by forming TNTs and mitochondrial transfer. (A-B)** Immunofluorescent staining revealed the transfer of mitochondria between the two cells, using MTR-hUCMSCs and MTG-MCs. Scale bars: 30 μm. **(C)** Flow cytometry to statistically analyze the proportion of MCs that have received mitochondria from hUCMSCs. **(D)** Schematic diagram of the composition of TNTs and the inhibitors of its corresponding components: Nocodazole (Noc, tubulin inhibitor), Cytochalasin D (CyD, F-actin inhibitor). **(E)** Observed the relationship between ATP levels of MCs and the formation of TNTs.** (F-G)** JC-1 assay for measuring mitochondria depolarization by immunofluorescent staining and microplate reader. Scale bars: 30 μm. **(H)** Apoptosis level of MCs before and after blocking the formation of TNTs. **(I)** Blocked the formation of TNTs and observed the oxidative stress levels by flow cytometry. Using Rosup as a positive control, calculated the relative fluorescence intensity of each group.** (J)** Confocal microscope detected the intracellular ROS level, and the green fluorescence intensity was summarized in the bar graph.** (K)** Microplate reader detected ROS level and calculated the relative ROS level using MCs as control.** (L)** Migration ability of MCs before and after blocking the formation of TNTs by transwell assay. Scale bars: 100 μm. **(M-N)** Observed the relationship between proliferative ability of MCs and the formation of TNTs: light microscope, CCK-8 assay. Scale bars: 150 μm. **(O-P)** Melanin content and the ability to synthesize melanin of MCs with or without TNTs blocking by NaOH cracking method and tyrosinase activity. Data show mean ± S.D, and each experiment was conducted with a minimum of three replicates. *P≤0.05, **P≤0.01, ***P≤0.001, ****P≤0.0001, by two-tailed Student's* t* test and one-way ANOVA with Bonferroni's post-test analysis.

MSCs can naturally transfer their mitochondria to various cell types, such as neurons, vascular endothelial cells, and lung alveolar epithelial cells, via TNTs. This process has been found to be crucial for the survival of the recipient cells 13, 14. However, the transfer of mitochondria from hUCMSCs to MCs within our co-culture system remains unexplored. To further identify the substances transmitted between cells via the structure of TNTs, we labeled the mitochondria of hUCMSCs and MCs with different mitoTracker and observed mitochondrial transfer after co-culturing the two cell types (Fig. 5A). The directionality of mitochondrial transfer between the two cells was birectional (Fig. S4B). While in our study, this kind of mitochondrial transfer was mainly appeared in CD117^+^ cell (Fig. S4C). Additionally, we observed that mitochondrial transfer initiated at 4 hours post co-culture of the two cell types and peaked around 8 hours, after which the number of MCs with mitochondria derived from hUCMSCs nearly ceased to increase (Fig. S4D). Flow cytometric and immunofluorescence analyses of hUCMSCs co-cultured with MCs, revealed that approximately 50% of the MCs contained MTR-labeled mitochondria after 8 hours *in vitro*, suggesting a transfer of mitochondria from the MTR-hUCMSCs (Fig. 5B-C).

To investigate whether hUCMSCs enhance the aforementioned functions of MCs by mediating mitochondrial transfer through the formation of TNTs between the two cells, we applied the tubulin inhibitor nocodazole (Noc) and F-actin inhibitor cytochrome D (CyD) to inhibit the formation of TNTs and observed its effects on the function of MCs co-cultured with hUCMSCs (Fig. 5D). We assessed the viability and mitochondrial transfer condition of MCs under various concentration gradients of Noc and CyD (Fig. S4F). The results indicated that treatment with 18.75 nM of either Noc or CyD blocked mitochondrial transfer (Fig.S5) without affecting cell viability (Fig. S6A-C). While this concentration would not influence the migration of MCs (Fig. S6D-E). Therefore, the subsequent use of these drugs at this concentration to block the formation of TNTs was recommended.

We then observed that in the presence of hUCMSCs, among the indicators related to mitochondrial function, the ATP synthesis level (Fig. 5E) and mitochondrial membrane potential of MCs increased (Fig. 5F-G), while the apoptosis rate (Fig. 5H) and the oxidative stress level (Fig. 5I-K) decreased. However, the effect of hUCMSCs was inhibited after blocking the formation of TNTs. Furthermore, we observed the function of MCs after blocking the formation of TNTs, and the results indicated that hUCMSCs had the ability to enhance the proliferation, migration, and melanin synthesis of MCs. This effect was inhibited by blocking the formation of TNTs (Fig. 5L-P).

In summary, TNTs mediated the enhancement of both mitochondrial function and melanocyte function of MCs in the presence of hUCMSCs.

### Inhibited TNT formation abolished the enhancement of MC functions by hUCMSCs

**Figure 6:**
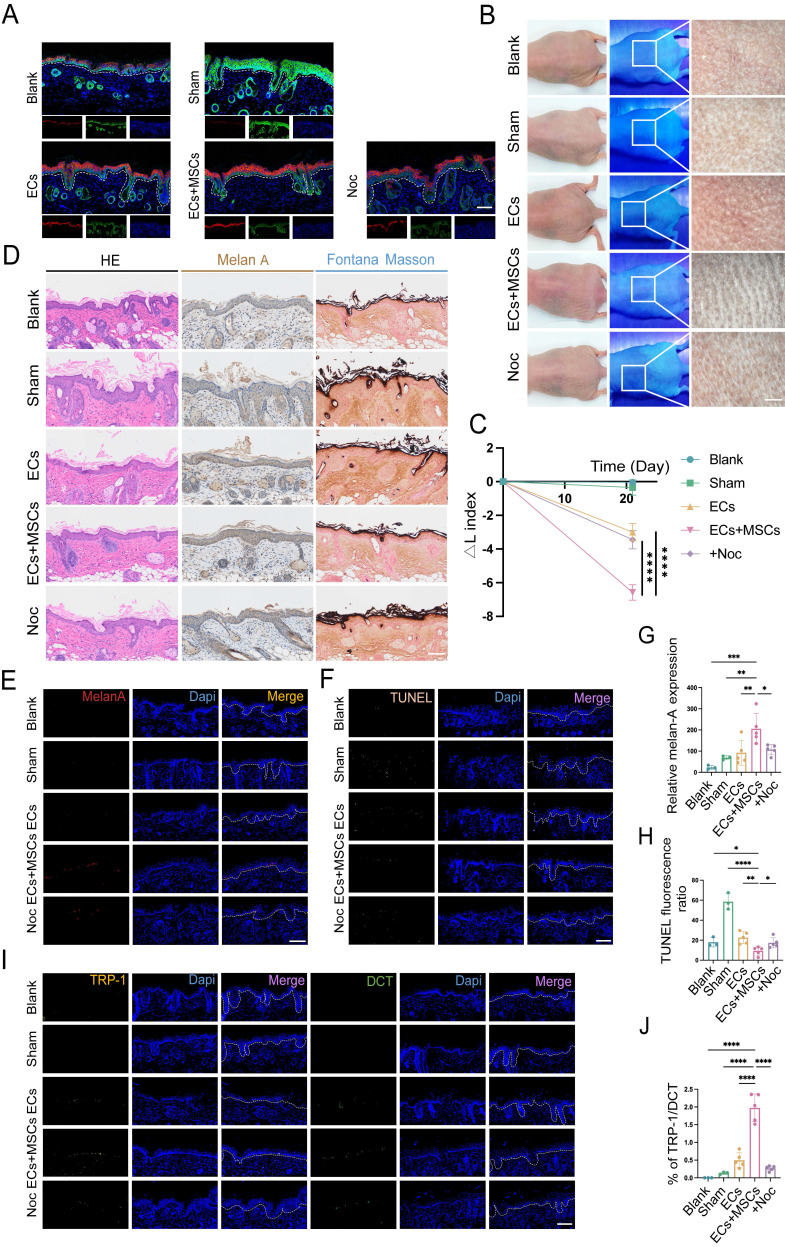
** TNTs mediate the enhancement of MCs functions by hUCMSCs. (A)** After transplantation, the source of ECs in the corresponding sites were represented by hu-K10 and hu/ms-K5. Scale bars: 100 μm.** (B)** After transplantation for 21 days, the transplantation sites were observed under white light, wood light, and skin microscope. Scale bars: 500 μm. **(C)** Changes in skin pigmentation were assessed spectrophotometrically and ΔL values were quantified before and after animal transplantation. **(D)** Light microscopy of skin specimens stained with H&E, Melan-A immunohistochemistry, and Fontana-Masson to compare the residing location of MCs and melanin content. Scale bars: 100 μm. **(E-F)** Immunofluorescence staining: melan-A, TUNEL apoptosis assay to detect the number, apoptosis condition of MCs. Scale bars: 100 μm.** (G-H)** Semi-quantitative analysis of the fluorescence intensity.** (I-J)** Immunofluorescence staining of TRP-1 and DCT to detect mature proportion of MCs. Scale bars: 100 μm. Data show mean ± S.D, and each experiment was conducted with a minimum of three replicates. *P≤0.05, **P≤0.01, ***P≤0.001, ****P≤0.0001, ns indicates no significant difference (n=3; two-tailed Student's *t* test).

To further verify the role of TNTs in AECS co-transplantation with hUCMSCs *in vivo*, we added 18.75 nM Noc to the suspension of AECS and hUCMSCs in the Noc group mice. After transplantation, the source of ECs at the transplantation sites was detected and the ECs in all transplant groups were primarily of human origin (Fig. 6A). Then the skin pigmentation was observed under white light, Wood's light, and with a skin microscope.

The results indicated that inhibiting the formation of TNTs, the skin color at the transplantation sites was lighter than that of the hUCMSCs and AECS co-transplantation group (Fig. 6B). It was confirmed by ΔL values to quantify the repigmentation condition (Fig. 6C). The distribution and melanin synthesis of MCs in transpanted sites were detected by H&E staining, Melan-A immunohistochemistry, and Fontana-Masson staining. The results indicated that inhibiting the formation of TNTs led to reduced colonization and melanin synthesis by MCs in the basal layer of mouse skin (Fig. 6D). Meanwhile, we utilized Melan-A fluorescence staining to conduct a quantitative analysis of the implanted MCs. The results indicated that after inhibiting the formation of TNTs, hUCMSCs exhibited a certain degree of inhibition on the color-enhancing effect of AECS (Fig. 6E, G). Finally, we utilized TRP-1/DCT staining and a TUNEL apoptosis assay to assess the functionality of the transplanted MCs. The results indicated that upon inhibiting the formation of TNTs, the colonization of functional MCs diminished, and the rate of MCs apoptosis appeared to rise (Fig. 6F, H-J).

In summary, the formation of TNTs and the subsequent transfer of mitochondria from hUCMSCs are essential for robust MCs function.

### TNFAIP2 regulates the formation of TNTs and enhances the function of MCs

**Figure 7:**
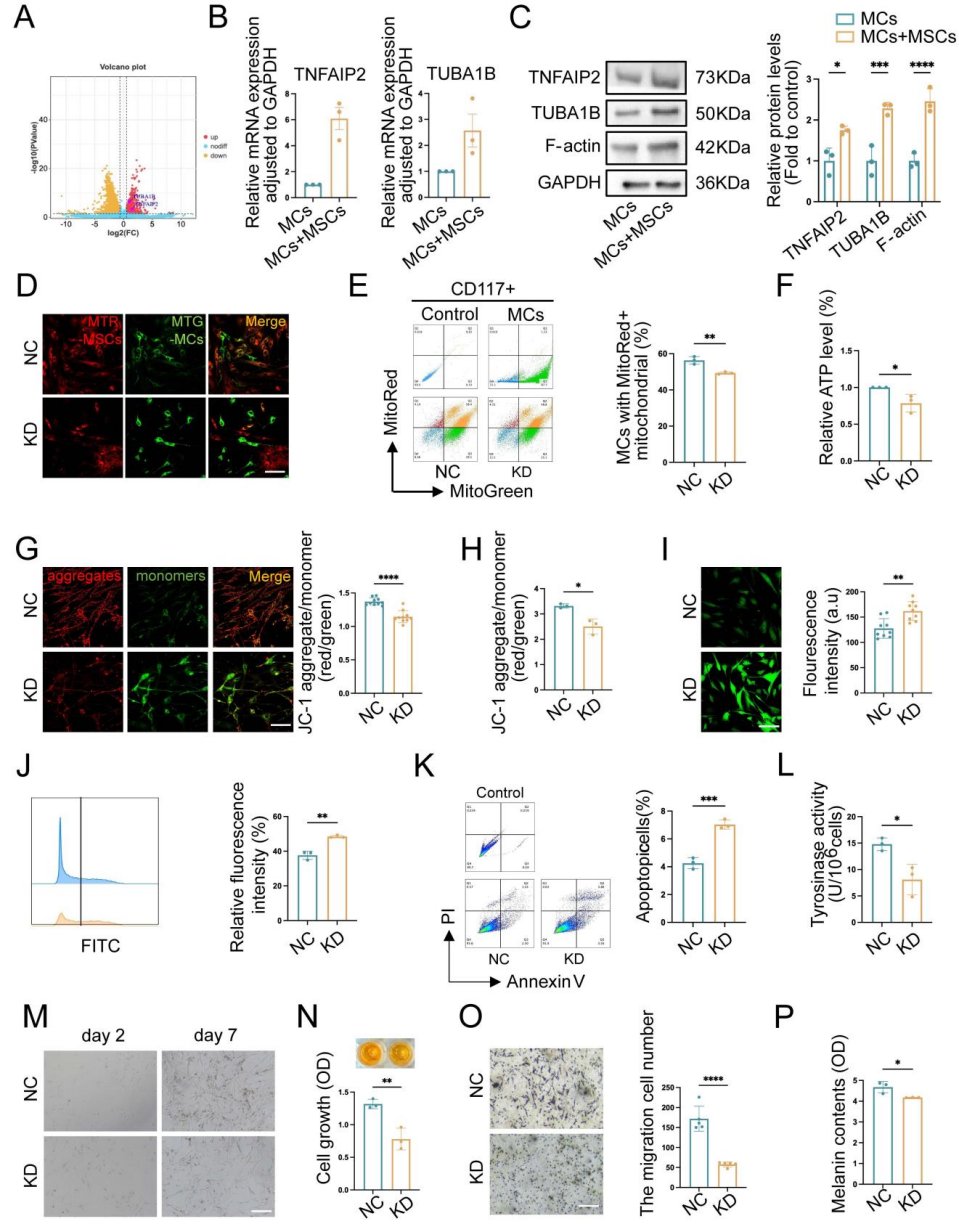
** TNFAIP2 knockdown inhibits mitochondrial transfer and the function of MCs. (A)** Volcano plot depicted the upregulation of TNTs regulating gene in co-MCs group. **(B-C)** Verified the target genes at the RNA and protein levels using qPCR and WB. **(D-E)** Immunofluorescence staining, and flow cytometry were used to statistically analyze the proportion of TNFAIP2-KD MCs that received mitochondria from hUCMSCs. Scale bars: 30 μm. **(F)** ATP levels in TNFAIP2-KD MCs co-cultured with hUCMSCs. **(G-H)** The JC-1 assay was used to measure mitochondrial depolarization through immunofluorescent staining and analysis with a microplate reader. Scale bars: 30 μm.** (I-J)** The oxidative stress levels in TNFAIP2-KD MCs by immunofluorescent staining and flow cytometry. Scale bars: 30 μm. **(K)** The apoptosis rates in TNFAIP2-KD MCs. **(L)** Assessment of melanin synthesis capability in TNFAIP2-KD MCs via Tyrosinase Activity Assay. **(M-N)** The proliferation status of TNFAIP2-KD MCs observed under a light microscope and assessed by CCK-8 assay. Scale bars: 100 μm.** (O)** The migration of TNFAIP2-KD MCs by transwell assay. Scale bars: 100 μm. **(P)** NaOH assay measuring the content of melanin changes in TNFAIP2-KD MCs. Data show mean ± S.D, and each experiment was conducted with a minimum of three replicates. *P≤0.05, **P≤0.01, ***P≤0.001, ****P≤0.0001, by two-tailed Student's *t* test.

Recent studies suggested that the representative function of TNFAIP2 included motivating the inflammatory response, promoting angiogenesis, facilitating cell proliferation, adhesion, migration, and inducing tunnel nanotube formation. TUBA1B was a key microtubule protein contributes significantly to cytoskeletal formation 31. A study of tumor revealed that TUBA1B regulated key cell cycle processes, driving tumor proliferation, migration, and invasion 32. There was increasingly recognized significance of them as a potential therapeutic target and immune response. To further investigate the regulatory mechanism of TNT formation in the co-cultured group, we conducted a differential gene analysis on RNA-seq sequencing data and discovered that *TNFAIP2* and *TUBA1B* were upregulated in MCs co-cultured with hUCMSCs (Fig. 7A). Therefore, we utilized qPCR and Western blotting (WB) to validate the aforementioned target genes, and the results were consistent with the sequencing outcomes (Fig. 7B-C).

**Figure 8:**
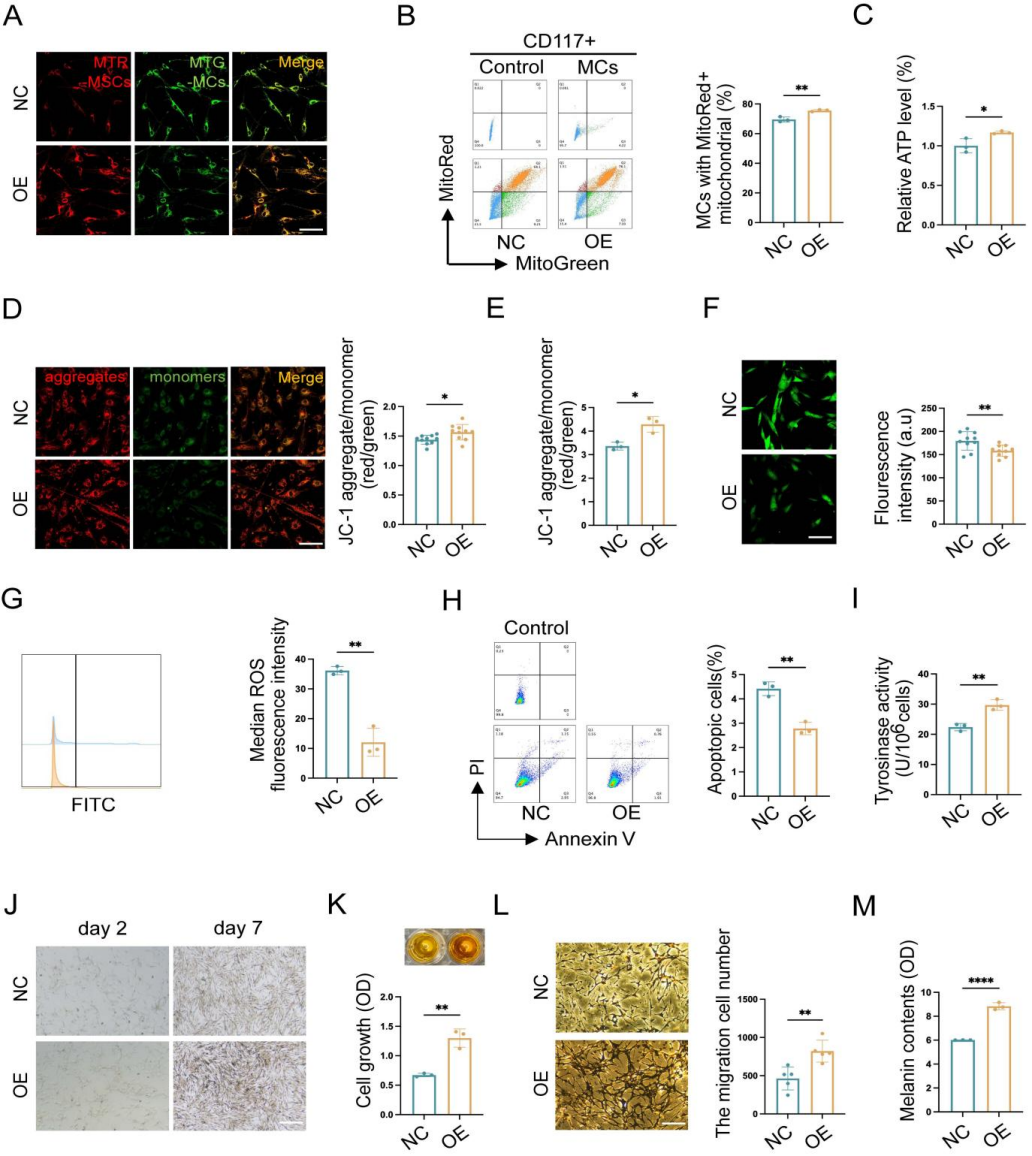
** TNFAIP2 overexpression enhances mitochondria transfer and the function of MCs. (A-B)** After treatment with TNFAIP2-OE plasmids, immunofluorescence staining, and flow cytometry were used to statistically analyze the proportion of MCs that had received mitochondria from hUCMSCs. Scale bars: 30 μm. **(C)** ATP levels in TNFAIP2-OE MCs co-cultured with hUCMSCs. **(D-E)** The JC-1 assay for measuring mitochondrial depolarization in TNFAIP2-overexpressing MCs co-cultured with hUCMSCs using immunofluorescent staining and a microplate reader. Scale bars: 30 μm.** (F-G)** The oxidative stress levels in TNFAIP2-OE MCs co-cultured with hUCMSCs by immunofluorescent staining and flow cytometry. Scale bars: 30 μm. **(H)** Apoptosis level in TNFAIP2-OE MCs after co-cultured with hUCMSCs. **(I)** Assessment of melanin synthesis capability in TNFAIP2-OE MCs via Tyrosinase Activity Assay. **(J-K)** After treatment with TNFAIP2-OE plasmids, the proliferation status of MCs was observed under a light microscope and assessed using a CCK-8 assay. Scale bars: 150 μm. **(L)** After treatment with TNFAIP2-OE plasmids, the migration of MCs was assessed using a transwell assay. Scale bars: 100 μm.** (M)** NaOH assay measuring the content of melanin changes in TNFAIP2-KD MCs. Data show mean ± S.D, and each experiment was conducted with a minimum of three replicates. *P≤0.05, **P≤0.01, ****P≤0.0001, by two-tailed Student's *t* test.

Mitochondria serve as an energy-supplying substance for cellular activities and play a crucial role in the survival and function of MCs 5, 7, 16. Therefore, the mitochondrial function after *TNFAIP2* knockdown and overexpression in MCs was detected. We co-cultured *TNFAIP2*-KD MCs and *TNFAIP2*-OE MCs with hUCMSCs separately, and then assessed the mitochondrial transfer status and the function of MCs. The results indicated that the mitochondrial transfer ratio was decreased in the *TNFAIP2*-KD MCs (Fig. 7D-E). Additionally, indicators characterizing mitochondrial function, such as cellular ATP levels and mitochondrial membrane potential were also found to be reduced (Fig. 7F-H). Conversely, the oxidative stress levels and cell apoptosis rates increased (Fig. 7I-K). Next, we examined the ability of MCs to proliferate, migrate, and synthesize melanin. The results indicated that the absence of *TNFAIP2* negated the enhancing effect of hUCMSCs on MC function (Fig. 7L-P). Conversely, overexpression of *TNFAIP2* resulted in an increase in the transfer of mitochondria from hUCMSCs to MCs (Fig. 8A-B) and enhanced the function of mitochondria in MCs (Fig. 8C-H). At the functional level of MCs, the overexpression of TNFAIP2 can enhance their ability to proliferate, migrate, and synthesize melanin under co-culture with hUCMSCs (Fig. 8I-M).

In conclusion, our findings suggest that TNFAIP2 promotes the proliferation, migration, and melanin synthesis of MCs by facilitating the polymerization of Tubulin and F-actin to form TNTs between hUCMSCs and MCs, which mediate the transfer of mitochondria from the former to the latter.

### TNFAIP2 promotes the expression of proteins associated with the formation of TNTs and the synthesis of melanin

**Figure 9:**
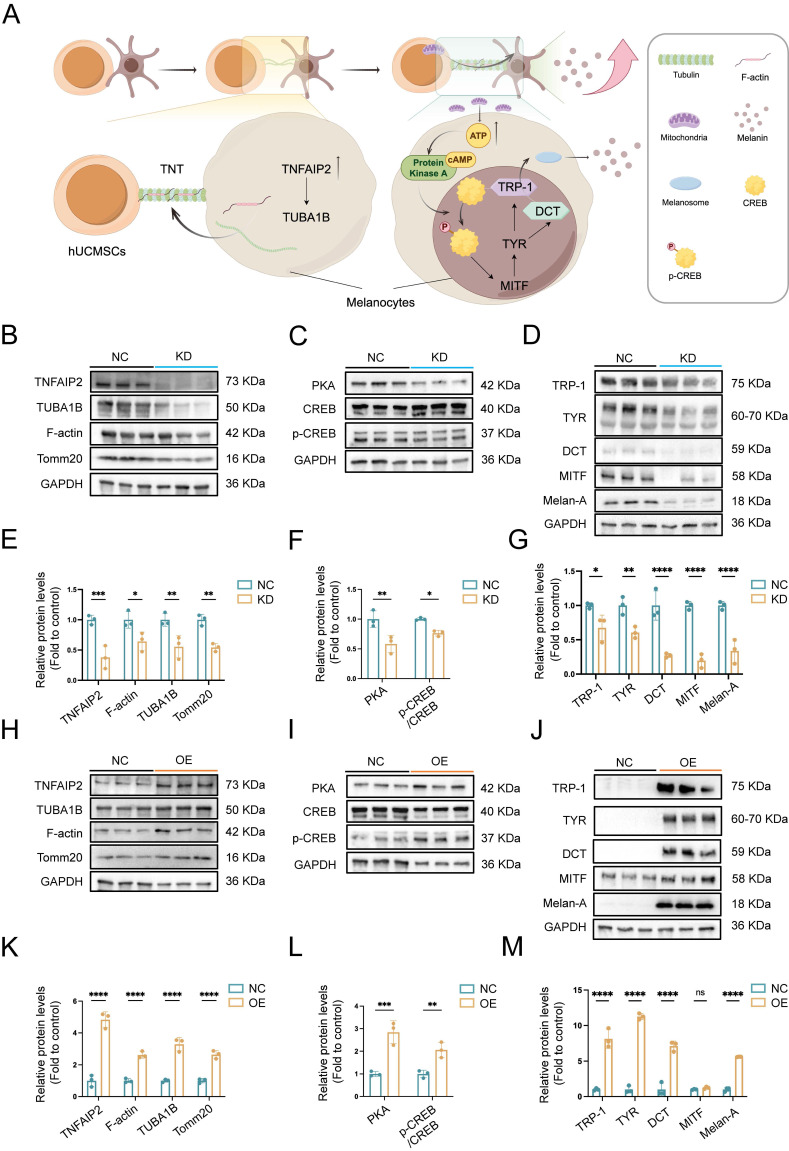
** TNFAIP2 regulates the expression of proteins associated with TNTs formation and the melanin synthesis. (A)** Mechanism diagram of TNFAIP2 regulating TNTs formation and improving melanin synthesis. **(B-D)** After the siTNFAIP2 treatment, the Western blot results indicated the expression levels of TNTs and mitochondria-related proteins, including TNFAIP2, TUBA1B, F-actin, and Tomm20, as well as proteins involved in the PKA/MITF pathway, such as PKA, CREB, p-CREB, and proteins associated with melanin synthesis and transfer, including TRP-1, TYR, DCT, MITF, and Melan-A. **(E-G)** Quantitative analysis of protein expression in TNFAIP2-KD MCs. **(H-J)** After treatment with TNFAIP2-OE plasmids, the Western blot results indicated the expression of the aforementioned proteins. **(K-M)** Quantitative analysis of protein expression in TNFAIP2-OE MCs. Data show mean ± S.D, and each experiment was conducted with a minimum of three replicates. *P≤0.05, **P≤0.01, ***P≤0.001, ****P≤0.0001, ns indicates no significant difference, by two-way ANOVA.

To further investigate the effects of *TNFAIP2* on the formation of TNTs and melanin synthesis, mitochondrial transmission mediated by TNTs and their relationship with melanin synthesis, we constructed *TNFAIP2* knockdown (*TNFAIP2*-KD) and *TNFAIP2* overexpression (*TNFAIP2*-OE) MCs respectively, then co-cultured them with hUCMSCs for WB detection (Fig. 9A). These were then co-cultured with hUCMSCs for 3 days, followed by Western Blot (WB) detection. The results indicated that the expression of proteins associated with TNT formation, including TUBA1B and F-actin, as well as the mitochondrial protein Tomm20, was diminished in *TNFAIP2*-KD MCs (Figure 9B). As a substrate for adenylate cyclase, ATP can further cause an increase in cAMP, thereby initiating the PKA/MITF pathway for melanin synthesis 33. Then we validated the PKA/MITF pathway on the co-culture group and the results indicated that the expression levels of PKA and the ratio of p-CREB/CREB were diminished in *TNFAIP2*-KD MCs (Fig. 9C). Finally, we extracted proteins to measure the expression levels of downstream melanin synthesis and transfer related genes. The results indicated that the expression levels of these proteins were diminished in *TNFAIP2*-KD MCs (Fig. 9D). Statistical analyses were confirmed with visual observations (Fig. 9E-G). Conversely, these proteins were elevated in *TNFAIP2*-OE MCs (Figure 9H-M).

These findings suggest that *TNFAIP2* regulates the formation of TNTs and melanin synthesis in MCs.

## Discussion

This study offers crucial insights into the role of the TNFAIP2-TNT system in enhancing the repigmentation efficacy of hUCMSCs co-transplanted with AECS, a key process in the development of AECS treatment. We demonstrate that TNFAIP2 regulates tubulin and F-actin in the cytoplasm and on membranes, facilitating the aggregation and formation of intercellular TNTs. This, in turn, modulates the transfer of mitochondria from hUCMSCs to MCs, thereby increasing the ATP levels in MCs. On the one hand, this causes an increase in ATP cyclic product cAMP, which significantly activates the cAMP/APK/MITF pathway, leading to increased levels of TRP1 and DCT. Consequently, TRP1 and DCT migrate out of the cell nucleus, causing melanoblasts to gradually differentiate into melanocytes and increasing the synthesis of melanin 34. On the other hand, ATP provides energy for cellular activity, enhancing the proliferation and migration of MCs, while inhibiting their apoptosis. Consistented with a study on photobiomodulation (PBM) therapy for vitiligo that improving mitochondrial function could increase ATP synthesis, enhance the proliferation and migration of MCs, and promote repigmentation 35. These findings clarify the molecular mechanisms that underlie the function of TNFAIP2 in the formation of TNTs and the up-regulation of genes related to melanin synthesis, which is the first to link TNTs, a physical organelle transfer, with cell-specific functions. This provides a novel theoretical foundation for utilizing TNFAIP2 to improve the repigmentation efficiency of AECS transplantation.

Compared with traditional treatment methods such as skin grafting, the AECS exhibits multiple advantages. These include the alleviation of postoperative pain, the reduction of postoperative scars, a shorter hospitalization period, the restoration of the original skin color, and cost - effectiveness. Additionally, it necessitates a smaller donor site area, indicating promising application prospects 2. Our research investigated the optimal conditions for enzymatic digestion in the preparation of AECS, utilizing cell yield and cell activity as evaluation criteria. These criteria represent the most crucial characteristics of cell therapy products, and our study fills the gap in the standardization of AECS preparation conditions 17, 36, 37. A clinical study carried out by Hoda M et al. on the impact of different trypsinization methods in vitiligo patients receiving non-cultured epidermal cellular suspension revealed that the highest cell viability among the three methods was 74.25 ± 18.80% 38. Additionally, a study by Liu Zhi et al. on the modification of the human skin cell isolation protocol demonstrated that the cell viability of epidermal cell suspension prepared via the traditional dispase/trypsin-EDTA protocol and TrypLE protocol was approximately 70% 17. The cell viability of AECS prepared under the optimal conditions we explored was 80.33 ± 2.39%. Moreover, the assessment of cell yield indicated that the yield of isolated epidermal cells was higher when our optimal conditions were employed compared to the traditional dispase/trypsin-EDTA protocol or TrypLE protocol (7.11±2.43×10^6^/cm^2^ vs. 4-5×10^5^/cm^2^) 17. Our study offers an improvement direction for the current clinical application of AECS transplantation therapy.

There were reports indicating that the repigmentation efficacy of AECS was restricted, and a variety of complications occurred, including hyperpigmentation, scarring, infections, milia formation, Koebner phenomenon, achromic fissures, and new depigmented patches 39. Previous research has demonstrated that the co-transplantation of endothelial colony-forming cells and MSCs induces the latter to express adhesive proteins. These adhesive proteins mediate intercellular and cell-extracellular matrix couplings, playing crucial roles in integrating exogenously delivered cells into the host tissue 40. In another investigation, it was discovered that MSCs enhanced the bioenergy, adaptability, and engraftment capacity of vascular endothelial cells through the transfer of mitochondria to these cells 12. Consistent with the findings of these researchers, our study demonstrated that hUCMSCs promoted the proliferation, migration, adhesion, and melanin synthesis of epidermal cells, while simultaneously suppressing their apoptosis. These effects effectively resolved the engraftment problem of AECS following transplantation, thereby enhancing the transplantation efficacy of AECS. Moreover, previous studies have demonstrated that MSCs can inhibit the migration of various immune cells and the release of inflammatory factors, thereby establishing a stable microenvironment 41, 42. Exosome derived from ADSCs exerted an ameliorating effect on skin hyperpigmentation, which further improved complications of AECS 43. This improvement in the microenvironment of the transplantation site helps to reduce the complications associated with current AECS transplantation and enables a more effective repigmentation effect. Our study offers a novel direction and perspective for the future development of AECS technology.

Furthermore, this study investigated the mechanism by which hUCSMCs enhance the repigmentation efficacy of AECS. Through RNA sequencing, genes associated with the cytoskeleton, microtubules, and cell-cell junctions were found to be upregulated. Subsequently, using electron microscopy and fluorescence techniques, we determined that this type of cell connection was TNTs. TNTs function as a novel mode of cell-cell communication, which plays a role in maintaining cellular homeostasis within the nervous system *in vivo*. Previous research has demonstrated that TNTs mediate mitochondrial transport and are essential for the development and maintenance of multicellular organisms 44-46. In this study, we examined the mitochondria-related indicators of MCs subsequent to the formation of TNTs. We discovered that the quantity of mitochondria increased, cellular ATP levels rose, and mitochondrial function was enhanced, which implies the transfer of functional mitochondria from hUCMSCs. Moreover, we observed that TNTs and the mitochondrial transfer they mediated played a crucial role in the repigmentation efficacy of the co-transplantation of hUCMSCs with AECS. Consistent with this study, TNTs are regarded as the primary cytoarchitecture for mitochondrial transportation 46, 47. These findings offer novel insights indicating that hUCMSCs transplantation can restore normal cellular function and salvage apoptotic cells in local tissue through TNTs-mediated functional mitochondrial transfer 48.

This study further investigated the upstream genes that govern the formation of TNTs. Experiments involving the knockdown of *TNFAIP*2 revealed inhibitory effects on TNT formation, mitochondrial transfer, and the function of MCs. Overexpression experiments also confirmed the regulatory role of *TNFAIP2* in TNT formation from a reverse perspective. Previous reports have demonstrated the crucial involvement of Miro1 in TNT-mediated mitochondrial transfer from bone marrow mesenchymal stem cells (BMSCs) and the protection of nucleus pulposus cells (NPCs) against mitochondrial dysfunction 30, 49. The TNFAIP2-TNT system also participates in maintaining the structural and functional integrity of the blood-brain barrier, repairing damaged hippocampal neurons (HT22 cells) and exerting a protective effect on autophagy in diabetic podocytes, although the specific mechanisms underlying these effects remain unclear 50-52. Our study elucidates the role of the TNFAIP2-TNT system in epidermal MCs and clarifies its mechanism in regulating melanin synthesis for the first time. Through functional mitochondrial transfer to MCs, cellular ATP levels and its cyclized products increase, leading to the phosphorylation of CREB, which activates the PKA/MITF pathway for melanin synthesis. These findings establish the molecular interactions among the TNFAIP2-TNT system, ATP, and the melanin synthesis process, offering in-depth understandings of the mechanism through which the co-transplantation of hUCMSCs improves repigmentation efficacy in the AECS for the treatment of depigmented disorders. Additionally, this understanding will facilitate the future development of novel AECS technologies in the field of cell transplantation therapy.

## Supplementary Material

Supplementary figures.

## Abbreviations

AECS: autologous epidermal cell suspension
hUCMSCs: human umbilical cord mesenchymal stem cells
TNFAIP2: tumor necrosis factor alpha-induced protein 2
TNTs: tunneling nanotubes
MCs: melanocytes
KCs: keratinocytes
ECs: epidermal cells
MMC: Mitomycin C
CCK-8: cell counting kit-8
PET: polyethylene terephthalate
PFA: paraformaldehyde
H&E: hematoxylin and eosin
IHC: immunohistochemistry
FPKM: fragments per kilobase of transcript per million fragments mapped
SEM: scanning electron microscope
MTG: mitotracker green
MTR: mitotracker red
Noc: Nocodazole
CyD: Cytochalasin D
ROS: reactive oxygen species
FITC: fluorescein isothiocyanate
PI: propidium iodide
KEGG: kyoto encyclopedia of genes and genomes
GO: Gene Ontology
