# Supplementary material for: Human umbilical cord mesenchymal stem cell delivery of mitochondria to melanocytes enhances skin repigmentation efficacy in autologous epidermal cell suspension transplantation through the TNFAIP2-TNT system
Source: Int J Biol Sci. 2026 Jan 30;22(4):2135–56. doi: 10.7150/ijbs.128719 (PMC12905636; doi:10.7150/ijbs.128719)
Supplement: Supplementary file 1 — Supplementary figures. [file ijbsv22p2135s1.pdf]

SUPPLEMENTARY MATERIALS

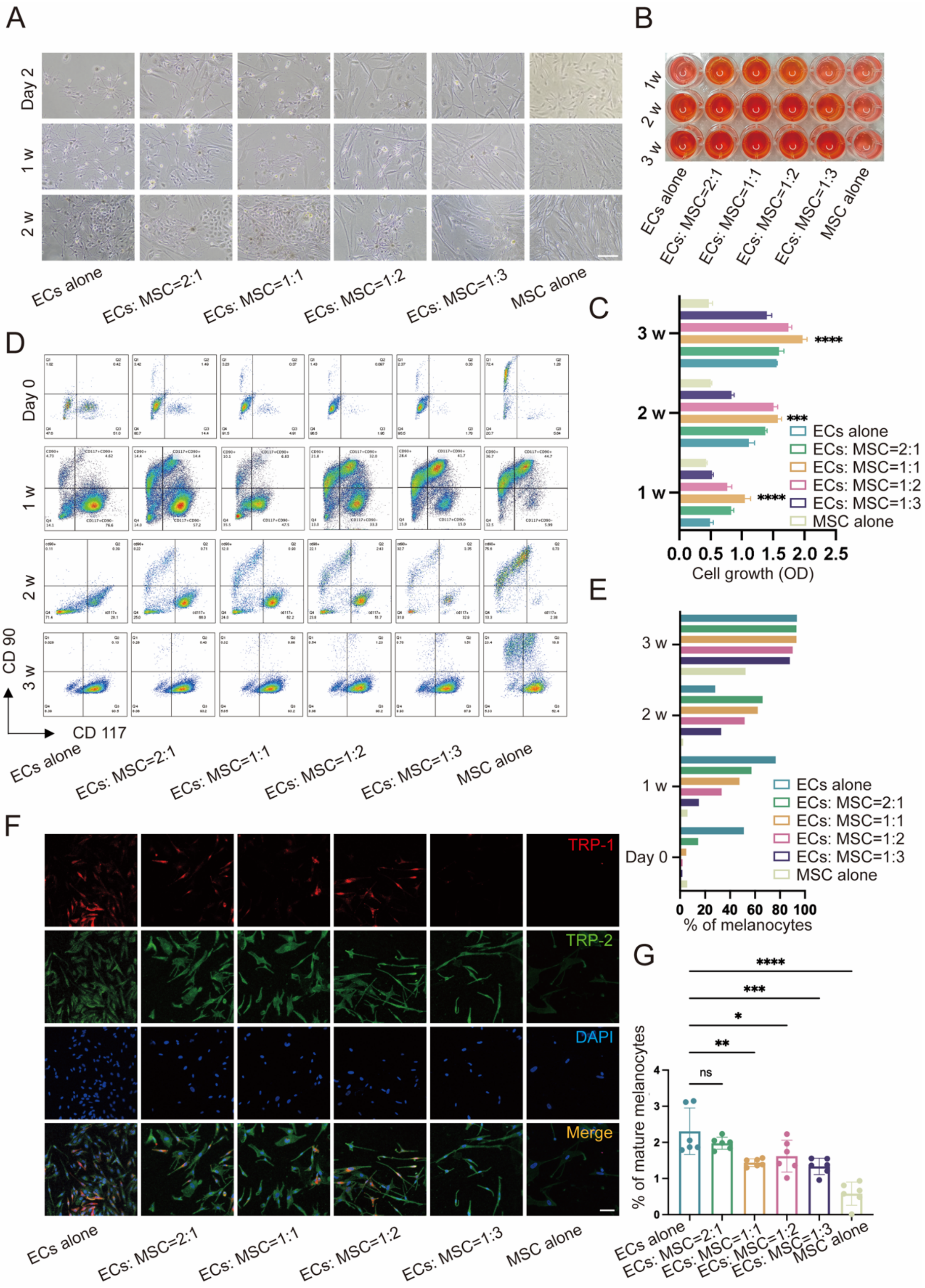

Figure S1. Proliferation and differentiation of ECs co-cultured with hUCMSCs at different ratios. (A) Observed the proliferation of ECs at different time points by light microscope. Scale bars: 100 μm. **(B)** Proliferative ability of ECs of co-culturing with hUCMSCs at different ratio by CCK-8. **(C)** Quantitative analysis of the proliferation of ECs. **(D)** Flow cytometry analyzed the percentage of MCs in the co-culture system. **(E)**

Immunofluorescent staining of MCs co-cultured with hUCMSCs at different ratios for 2 weeks suggested the differentiation situation of MCs. Scale bars: 30 μm. Data show mean ± S.D, *P≤0.05, **P≤0.01, ***P≤0.001, ****P≤0.0001.

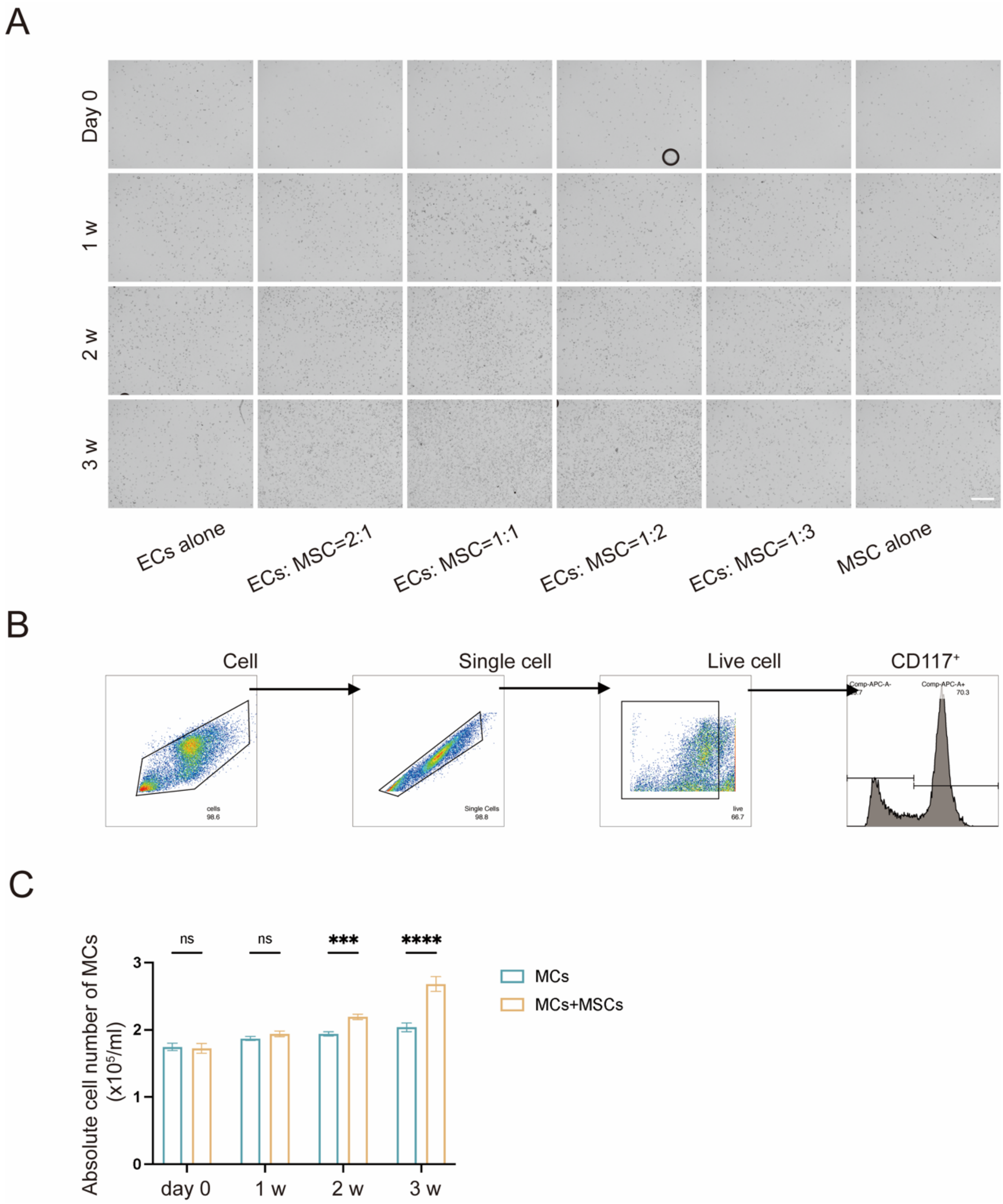

Figure S2. The proliferation of MCs in ECs alone and co-culture with hUCMSCs. (A) Cell count on ECs and co-cultured ECs with hUCMSCs at different time points. Scale bars: 200 μm. **(B)** Gating strategy of CD117^{+} marked MCs after co-culture by flow cytometry. **(C)** The absolute cell number of MCs calculated with cell count and flow cytometry. Data show mean ± S.D, and each experiment was conducted with a minimum of three replicates. ***P≤0.001, ****P≤0.0001, ns indicates no significant difference by two-way ANOVA.

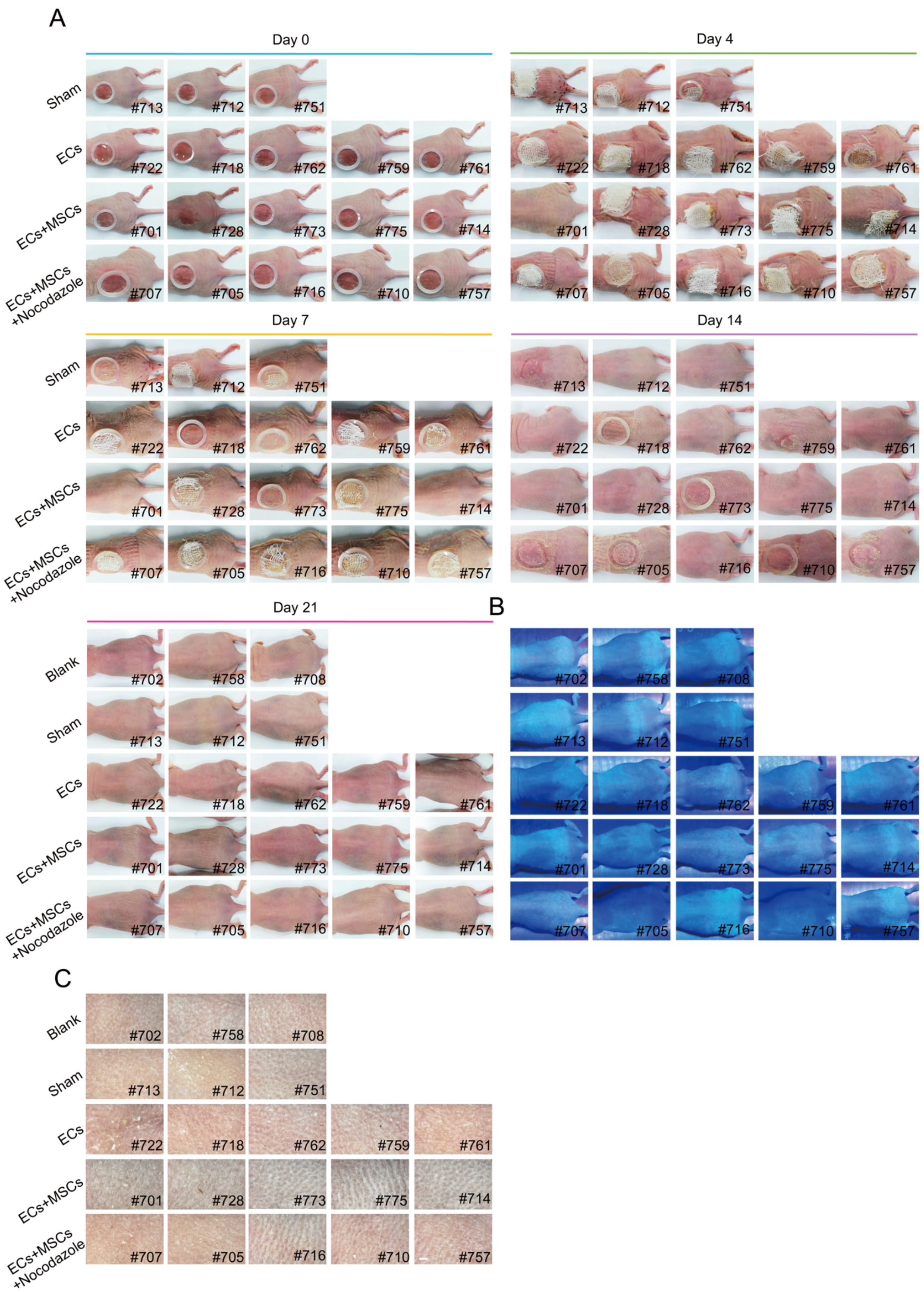

Figure S3. Skin growth and repigmentation on the dorsal side of mice at different time points after transplantation. (A) Observed the repigmentation condition of mice at different time points. **(B)** Wood light analyzed the repigmentation condition of transplantation sites. **(C)** Skin microscope observed the pigmentation at the transplantation sites. Scale bars: 500 µm.

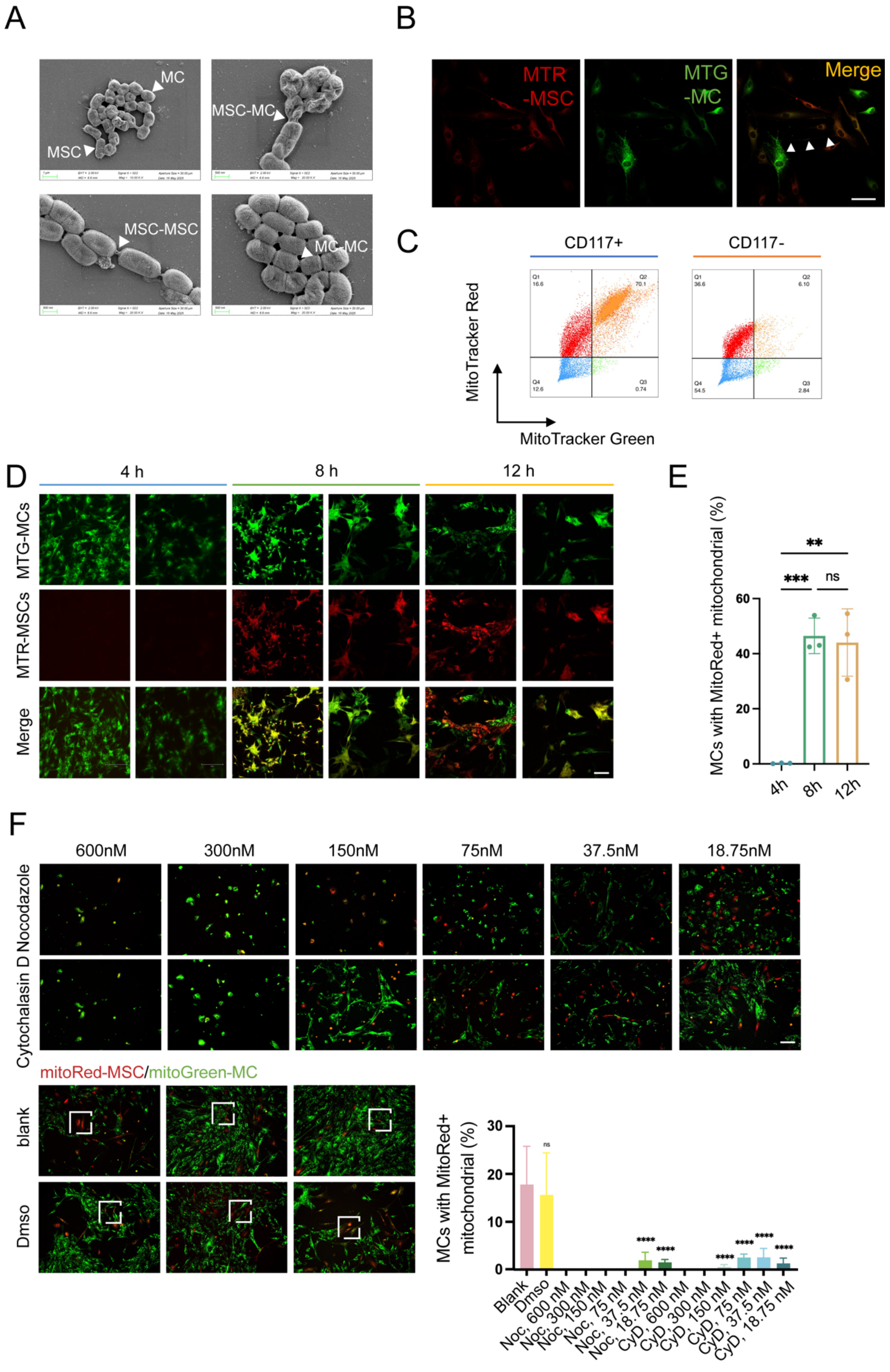

Figure S4. Observation of TNTs and selection of blocker concentrations. (A) Observation of three types of TNTs by SEM: MSC-MC, MSC-MSC and MC-MC. **(B)**

Observation of the coexistence of MTG and MTR in the same TNT between MC and MSC using confocal microscopy. Scale bars: 30 μm. **(C)** Flow cytometry detected the ratio of MTR and MTG in CD117+and CD117- cells. **(D)** Immunofluorescent staining revealed the transfer of mitochondria between the two cells using MTR-hUCMSCs and MTG-MCs at different time points. Scale bars: 30 μm. **(E)** Statistically analysis of MCs with MTR. **(F)** Immunofluorescent staining observed the blocking status of mitochondrial transfer following treatment with different drug concentrations of Noc or CyD for 7 days. Scale bars: 30 μm. Data show mean ± S.D, $^{**}P \leq 0.01$, $^{***}P \leq 0.001$, ns indicates no significant difference.

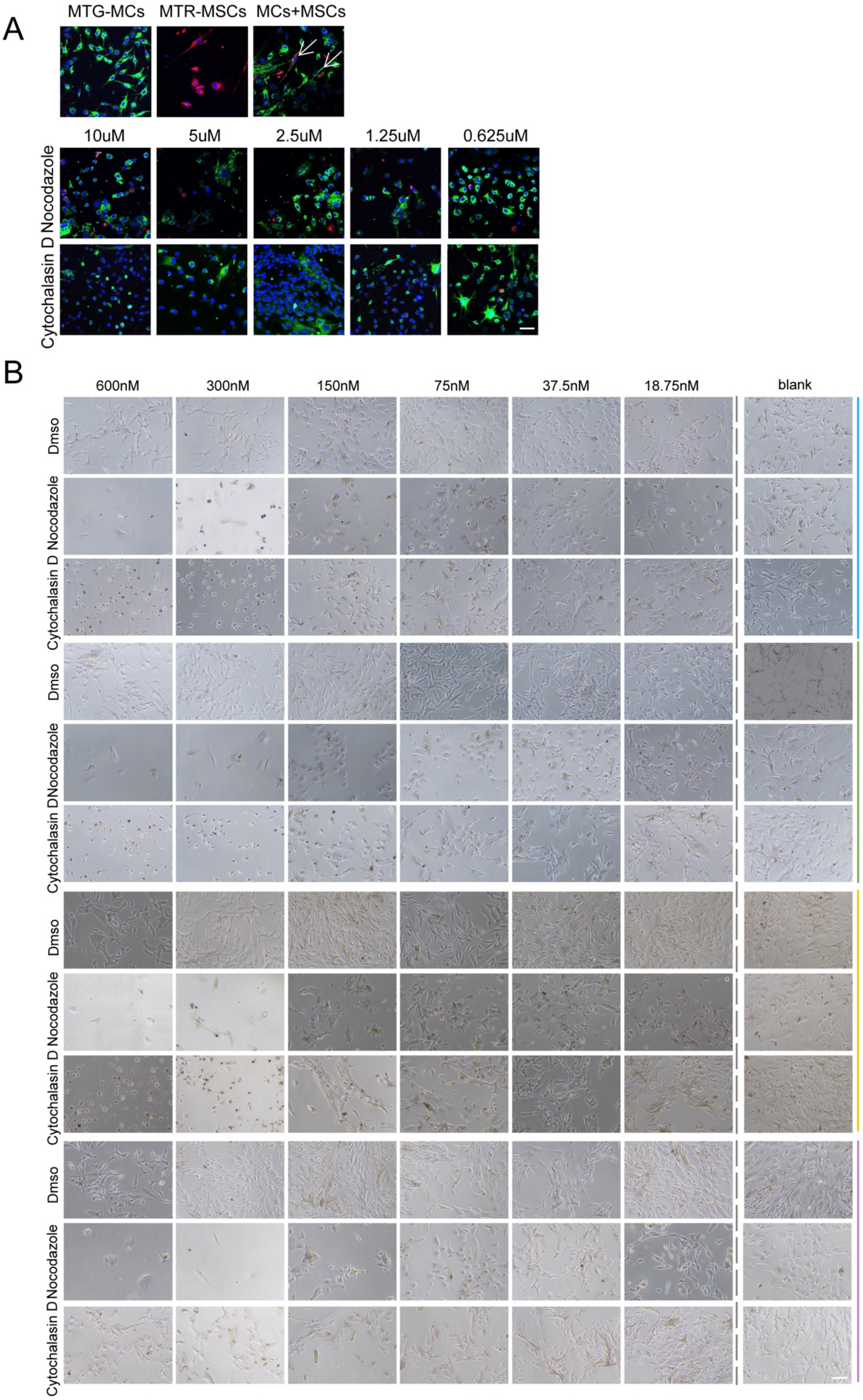

A
MTG-MCs
MTR-MSCs
MCs+MSCs
10uM
5uM
2.5uM
1.25uM
0.625uM
Nocodazole
Cytochalasin D
B
600nM
300nM
150nM
75nM
37.5nM
18.75nM
blank
Dmso
Nocodazole
Cytochalasin D
Day 2
Day 4
Day 6
Day 7

37

Figure S5. Proliferation of MCs treated with different concentrations of TNT inhibitor. (A) Immunofluorescent staining observed mitochondrial transfer following treatment with different drug concentrations of Nocodazole (Noc) or Cytochalasin D (CyD) for 8 hours. Scale bars: 30 μm. **(B)** Light microscope revealed the proliferation of ECs following treatment with different drug concentrations of Noc or CyD at different time points. Scale bars: 100 μm.

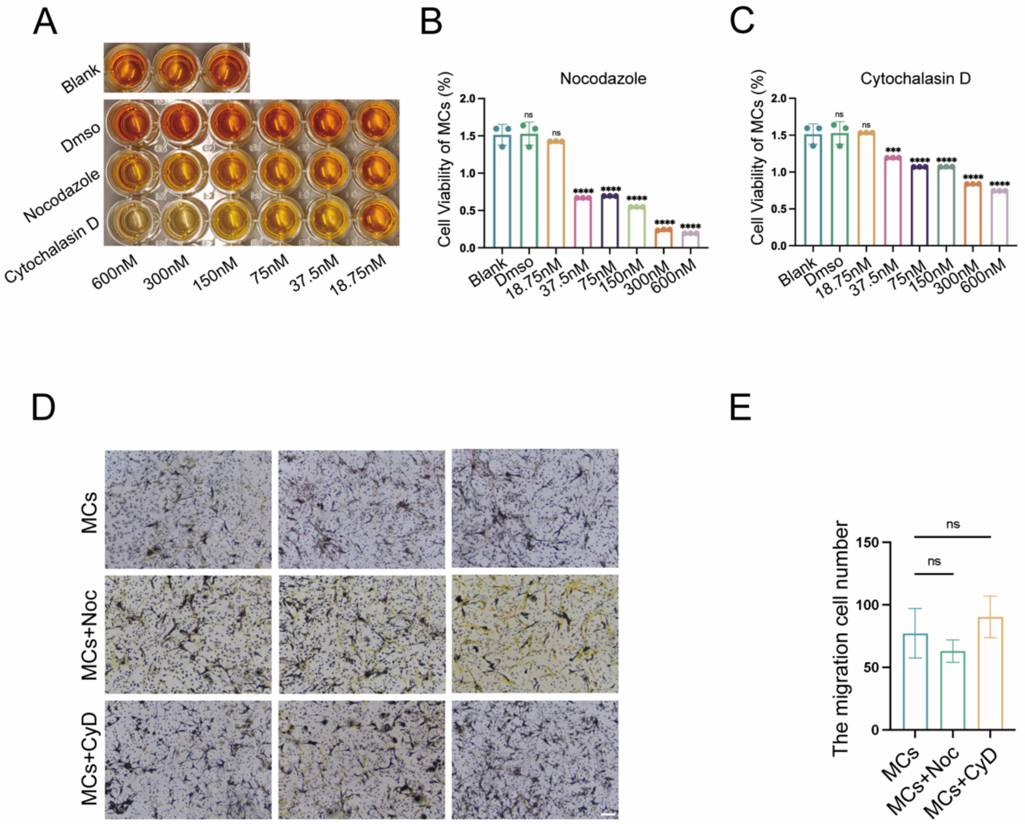

Figure S6. Proliferation and migration of MCs treated with 18.75 nM TNT inhibitor. (A-C) CCK8 revealed the proliferation of ECs following treatment with different drug concentrations of Noc or CyD at different time points. **(D)** Migration ability of MC compared with 18.75 nM Nocodazole or Cytochalasin D treated MCs at 72 h. Scale bars: 100 μm. **(E)** Statistically analysis of migration cell number. Data show mean ± S.D., ***P≤0.001, ****P≤0.0001, ns indicates no significant difference.
